# Environmental toxicants in breast milk of Norwegian mothers and gut bacteria composition and metabolites in their infants at 1 month

**DOI:** 10.1186/s40168-019-0645-2

**Published:** 2019-02-27

**Authors:** Nina Iszatt, Stefan Janssen, Virissa Lenters, Cecilie Dahl, Hein Stigum, Rob Knight, Siddhartha Mandal, Shyamal Peddada, Antonio González, Tore Midtvedt, Merete Eggesbø

**Affiliations:** 1 Department of Environmental Exposure and Epidemiology, Infection Control and Environmental Health, Norwegian Institute of Public Health, PO Box 222, Skøyen, 0213 Oslo, Norway; 2 Department of Pediatrics, University of California San Diego, 9500 Gilman Drive, La Jolla, CA 92093 USA; 3 Department of Pediatric Oncology, Hematology and Clinical Immunology, Heinrich-Heine University Dusseldorf, Dusseldorf, Germany; 4 Department of Community Medicine and Global Health, University of Oslo, Kirkeveien 166, Fredrik Holsts hus, 0450 Oslo, Norway; 5 Department of Non-communicable Disease, Norwegian Institute of Public Health, PO Box 222, Skøyen, 0213 Oslo, Norway; 6 Center for Microbiome Innovation, University of California San Diego, 9500 Gilman Drive, La Jolla, CA 92093 USA; 7 Department of Computer Science and Engineering, University of California San Diego, 9500 Gilman Drive, La Jolla, CA 92093 USA; 8 Public Health Foundation of India, Delhi NCR, Plot No. 47, Sector 44, Institutional Area Gurgaon, Gurgaon 122002, India; 9 Biostatistics Branch, National Institute of Environmental Health Sciences (NIEHS), 111 T.W. Alexander Drive, Durham, NC 27709 USA; 10 Department of Microbiology, Tumor and Cell Biology, Karolinska Institute, Nobels väg 16, Solna Campus, Box 280, SE-171 77 Stockholm, Sweden

**Keywords:** Breast milk, Toxicants, Short-chain fatty acids, Infant gut microbiome, Birth cohort

## Abstract

**Background:**

Early disruption of the microbial community may influence life-long health. Environmental toxicants can contaminate breast milk and the developing infant gut microbiome is directly exposed. We investigated whether environmental toxicants in breastmilk affect the composition and function of the infant gut microbiome at 1 month. We measured environmental toxicants in breastmilk, fecal short-chain fatty acids (SCFAs), and gut microbial composition from 16S rRNA gene amplicon sequencing using samples from 267 mother-child pairs in the Norwegian Microbiota Cohort (NoMIC). We tested 28 chemical exposures: polychlorinated biphenyls (PCBs), polybrominated flame retardants (PBDEs), per- and polyfluoroalkyl substances (PFASs), and organochlorine pesticides. We assessed chemical exposure and alpha diversity/SCFAs using elastic net regression modeling and generalized linear models, adjusting for confounders, and variation in beta diversity (UniFrac), taxa abundance (ANCOM), and predicted metagenomes (PiCRUSt) in low, medium, and high exposed groups.

**Results:**

PBDE-28 and the surfactant perfluorooctanesulfonic acid (PFOS) were associated with less microbiome diversity. Some sub-OTUs of *Lactobacillus*, an important genus in early life, were lower in abundance in samples from infants with relative “high” (> 80th percentile) vs. “low” (< 20th percentile) toxicant exposure in this cohort. Moreover, breast milk toxicants were associated with microbiome functionality, explaining up to 34% of variance in acetic and propionic SCFAs, essential signaling molecules. Per one standard deviation of exposure, PBDE-28 was associated with less propionic acid (− 24% [95% CI − 35% to − 14%] relative to the mean), and PCB-209 with less acetic acid (− 15% [95% CI − 29% to − 0.4%]). Conversely, PFOA and dioxin-like PCB-167 were associated with 61% (95% CI 35% to 87%) and 22% (95% CI 8% to 35%) more propionic and acetic acid, respectively.

**Conclusions:**

Environmental toxicant exposure may influence infant gut microbial function during a critical developmental window. Future studies are needed to replicate these novel findings and investigate whether this has any impact on child health.

**Electronic supplementary material:**

The online version of this article (10.1186/s40168-019-0645-2) contains supplementary material, which is available to authorized users.

## Background

During a critical period for developmental programming, infants may be exposed to both environmental toxicants and multiple microbiome-altering factors. Breastmilk is a unique and complex substance that has evolved to provide nutrition and crucial immune support at a time when the gut microbiome changes from low diversity at birth to a rapidly evolving ecosystem [1]. Breastfeeding has clear health benefits such as protection from infections, particularly in low- and middle-income countries [2]. However, breastmilk can also be contaminated with persistent toxicants [3], which may directly affect health or indirectly through interaction with the gut microbiome. These chemicals once commonly used in agriculture as pesticides and fungicides, and in manufacturing, as flame retardants or non-stick substances, are persistent organic pollutants, which bind to lipids or proteins and thus bioaccumulate and biomagnify through the food chain and are ubiquitous in the environment and human food such as fish, meat, and dairy [4, 5]. The maternal body burden of these toxic chemicals accumulates during her lifetime and is transferred to her baby in utero and through breastfeeding [6, 7]. These chemicals can affect immune, endocrine, and metabolic systems in humans, particularly following in utero exposure [8, 9]. Experimentally, some chemicals have been shown to alter the gut microbiome, although many studies were at high doses, with the effect of environmentally relevant doses less clear [10, 11].

Early toxicant exposure and the development of the gut microbiome occur during critical windows for developmental programming and immune system maturation [12], influencing later health [8, 13, 14]. Short-chain fatty acids (SCFAs) are signaling molecules primarily produced by gut microbiota during fermentation of non-digestible fibers and protein and are immunomodulatory [15] and neuromodulatory [16]. Emerging evidence suggests that neurodevelopment and metabolic disorders are associated with both less microbiome diversity [17] and chemical exposure [18, 19].

We investigated whether exposure to multiple environmental toxicants that are globally present in breastmilk [3, 20] is associated with gut microbiome composition and function among infants at 1 month.

## Results

### Characteristics of the study cohort

NoMIC is a Norwegian prospective birth cohort, where mothers were recruited in Østfold county hospital, two term for every preterm delivery [21–23]. Our analyses included 267 mother-child pairs where at 1-month postpartum both infant gut microbiota and breastmilk concentrations of chemicals have been characterized. Twins and infants who had antibiotics 2 weeks prior to sampling were excluded from the analyses. Mothers had a mean age of 30.4 (± 4.4) years, with a normal body mass index (mean 24.3 ± 4.5 kg/m^2^), were non-smokers (90%), educated more than 12 years (72.8%) and Caucasian (99.6%), and with  22.5% preterm deliveries due to the oversampling scheme (Additional file 1: Table S1). Table 1 shows the distributions of toxicants measured in this study. Overall, toxicants were detected in more than 96% of samples, with the exception of PBDE-154 (11.2% < LOD). In particular, there were low concentrations and variability of the polybrominated diphenyl ethers (PBDEs). There were statistically significant correlations within and between classes of toxicants. The highest cross-class correlations were between organochlorine pesticides and polychlorinated biphenyls (PCBs) (46% of the correlations *r* = 0.6 to 0.85), while flame retardant PBDEs and per- and polyfluoroalkyl substances (PFASs) were not strongly correlated with other toxicant classes (*r* < 0.56) (Additional file 1: Figure S1). Child fecal samples were characterized by 16S rRNA gene amplicon sequencing of the V4 region. We used Deblur, a novel sub-operational taxonomic-unit (sub-OTU) approach that provides a higher resolution than OTU-based analyses [24]. At 1 month, infants had a median Shannon diversity index of 2.3 (IQR 1.8–2.7) (Additional file 1: Table S2), and their gut microbiome was dominated by *Bifidobacterium*, followed by *Streptococcus*, *Erwinia*, and *Bacteriodes* (Additional file 1: Figure S2).

**Table 1:** Distribution of environmental chemicals in breast milk at 1 month post-partum (31.4 [± 19.9] days)

Class	Exposure	*N*	% missing^a^	% < LOD	Mean (± SD)	Min	p25	p50	p75	Max
Dioxin-like PCBs	PCB-105	266	0.3	0	1.64 (1.09)	0.41	0.99	1.4	1.93	12.72
	PCB-114	266	0.3	1.4	0.39 (0.22)	LOD	0.26	0.35	0.48	2.09
	PCB-118	266	0.3	0	7.68 (5.01)	1.99	4.96	6.82	8.95	62.23
	PCB-156	266	0.3	0	3.83 (2.34)	0.58	2.43	3.34	4.53	22.73
	PCB-157	266	0.3	0	0.76 (0.55)	0.09	0.44	0.61	0.9	4.87
	PCB-167	264	1	0	0.97 (0.65)	0.19	0.64	0.84	1.13	8.05
	PCB-189	266	0.3	0.3	0.30 (0.21)	LOD	0.19	0.25	0.35	2.48
Non-dioxin-like PCBs	PCB-74	266	0.3	0	4.12 (2.35)	0.84	2.65	3.74	4.8	18.68
	PCB-99	266	0.3	0	4.98 (2.66)	0.84	3.35	4.48	6.14	24.74
	PCB-138	266	0.3	0	22.63 (12.85)	4.39	15.54	20.27	26.89	145.06
	PCB-153	266	0.3	0	37.73 (22.50)	7.34	25.81	34.7	44.12	296.03
	PCB-170	266	0.3	0	7.79 (4.44)	1.22	5.25	6.89	9.27	46.49
	PCB-180	266	0.3	0	19.65 (12.05)	4.2	13.17	17.73	23.38	142.46
	PCB-194	266	0.3	0.3	1.56 (1.10)	LOD	0.94	1.37	1.85	11.76
	PCB-209	225	14.2	3.7	0.12 (0.10)	LOD	0.07	0.1	0.13	0.78
Organochlorine pesticides	HCB	266	0.3	0	11.14 (4.72)	1.72	8.72	10.45	12.93	48.72
	β-HCH	266	0.3	0	4.70 (2.98)	0.7	2.9	4.32	5.63	31.34
	*p,p*′-DDE	266	0.3	0	66.03 (54.00)	5.37	38.08	53.48	77.16	617.26
	*p,p*′-DDT	219	16.2	0.4	2.65 (2.80)	0.04	1.54	2.11	2.93	35.16
	Oxychlordane	258	3	0	3.77 (2.69)	0.53	2.38	3.27	4.48	30.27
PDBEs	PBDE-28	257	3.4	1.4	0.25 (0.47)	0.01	0.11	0.16	0.26	5.59
	PBDE-47	257	3.4	0	1.99 (4.96)	0.18	0.74	1.1	1.74	59.19
	PBDE-99	257	3.4	0	0.48 (1.18)	0.04	0.18	0.26	0.42	14.99
	PBDE-100	257	3.4	0.4	0.41 (0.74)	0.01	0.19	0.26	0.4	7.5
	PBDE-153	256	3.7	0	0.61 (0.48)	0.05	0.35	0.5	0.7	4.03
	PBDE-154	257	3.4	11.2	0.04 (0.09)	0	0.02	0.03	0.04	1.18
PFAS	PFOA	230	12.5	2.8	57.60 (33.98)	2.19	34.42	50.77	71.18	182.55
	PFOS	230	12.5	0	126.70 (63.07)	22.99	80.39	116.73	158.05	370.63

Breastmilk concentrations in ng/g lipid except for PFOS and PFOA, which are ng/L

*LOD* limit of detection, *PCB* polychlorinated biphenyl, *HCB* hexachlorobenzene, *β*-*HCH* beta-hexachlorocyclohexane, *p,p*′-*DDE* dichlorodiphenyldichloroethylene, *p,p*′-*DDT* dichlorodiphenyltrichloroethane, *PBDE* polybrominated diphenyl ether, *PFOA* perfluorooctanoic acid, *PFOS* perfluorooctanesulfonic acid

^a^“Missing” because the breast milk samples had not undergone chemical analysis at the time of this study. Only compounds that had *N* > 200 and > 80% of samples above the LOD were included

### PFOS and PBDE-28 associate with infant gut microbiome α-diversity

To test which of the 28 chemicals were associated with α-diversity (Shannon diversity, Faith’s phylogenetic diversity, and number of observed sub-OTUs), we used elastic net regression, a penalized regression method to select among correlated chemicals [25, 26], adjusting for C-section, preterm delivery, maternal α-diversity 4 days after birth, and proportion of meals given through breastfeeding at 1 month. Elastic net selected 2 of 28 toxicants as the best predictors associated with less α-diversity, although more PBDEs were associated in single pollutant models (Additional file 1: Figure S3). In the unpenalized model, a one standard deviation (SD) increase of 0.5 ng/g milk lipid of PBDE-28 was associated with less Shannon diversity (− 4%, 95% confidence interval [CI]: − 7% to − 2%, relative to mean Shannon), explaining 2% of variance. In the same model, preterm delivery was associated with less Shannon diversity (− 15%, 95% CI − 23% to − 7%), any formula feeding with more (11%, 95% CI − 1% to 24%), while none of the other potential confounders were associated. A 1SD increase of 63 ng/L of perfluorooctane sulfonate (PFOS) was associated with less phylogenetic diversity (− 5%, 95% CI − 9 to − 1%), explaining 4% of variance. Of the potential confounders in that model, only C-section was significantly associated with phylogenetic diversity (− 9%, 95% CI − 18% to − 1%). PFOS was also associated with number of observed sub-OTUs (− 7%, 95% CI − 12 to − 1%) (Additional file 1: Figure S3).

Sensitivity analyses (restricting to complete case, breast milk sample collection age < 60 days, exclusive breastfeeding, and excluding extreme values) attenuated effect estimates or altered precision in some cases, but in general did not affect the overall interpretation. When restricting to term births, elastic net additionally selected PCB-167 associated with significantly less phylogenetic diversity and number of sub-OTUs (Additional file 1: Table S5). We found no material influence on results from the inclusion/exclusion of additional variables or interactions (including preterm delivery) as described in the methods.

### PFOS and PCB-167 associate with infant gut microbiome β-diversity

**Fig. 1:**
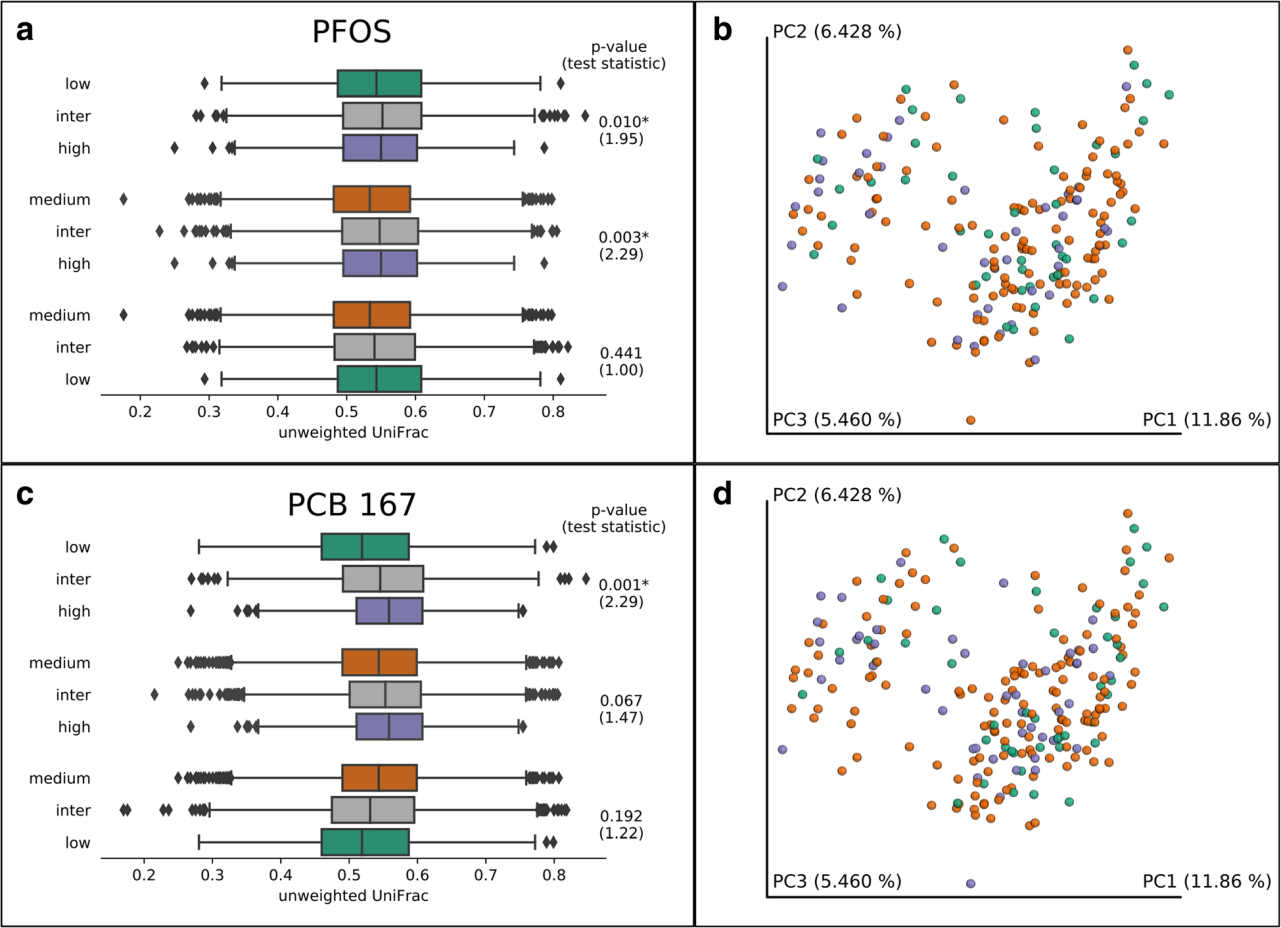
Environmental chemicals in breastmilk associate with infant gut microbiome β-diversity at 1 month by low, medium, and high exposure groups. PFOS and unweighted UniFrac, **a** box plots and **b** PCoA plot. PCB-167 and unweighted UniFrac, **c** boxplots and **d** PCoA plot. *P* value from PERMANOVA, * indicates significant after Bonferroni correction. There are three groups of boxplots per pairwise test showing distances *within* each of two exposure groups (“low”/“high,” “medium”/“high,” or “medium”/“low”) and “inter” the distances *between* exposure groups, e.g., **a**) The “inter” (gray) UniFrac distance between “low” PFOS exposed group (green) and “high” exposed group (purple) is greater than the distances within either the low group or the high group. Samples of both clusters partially overlap, obscuring these differences in PCoA plot B. In (**c**), the distances within the “low” (green) PCB-167 are lower (more similar) than the distances in the “high” (purple) group

For each toxicant, we tested whether infants exposed to “low” (< 20th), “medium” (≥ 20–≤ 80th) or relative “high” (> 80th percentile) breastmilk concentrations were more or less similar based on β-diversity distances (weighted or unweighted UniFrac), using permutational multivariate analysis of variance (PERMANOVA) to test significance, with Bonferroni correction for multiple testing. There were significant differences in community composition between PFOS exposure groups, with greater dissimilarity between high and medium exposed (*p* = 0.003), and between high and low exposed (*p =* 0.010) using unweighted UniFrac (Fig. 1a). Community composition in infants relatively highly exposed to PCB-167 was more diverse than in low exposed infants (*p* = 0.001) with unweighted UniFrac (Fig. 1c). There were no other significant differences in unweighted UniFrac, nor any differences in weighted UniFrac among other toxicant exposures.

### Relative abundance of sub-OTUs belonging to Firmicutes differ in infants in the “high” toxicant exposure group

**Fig. 2:**
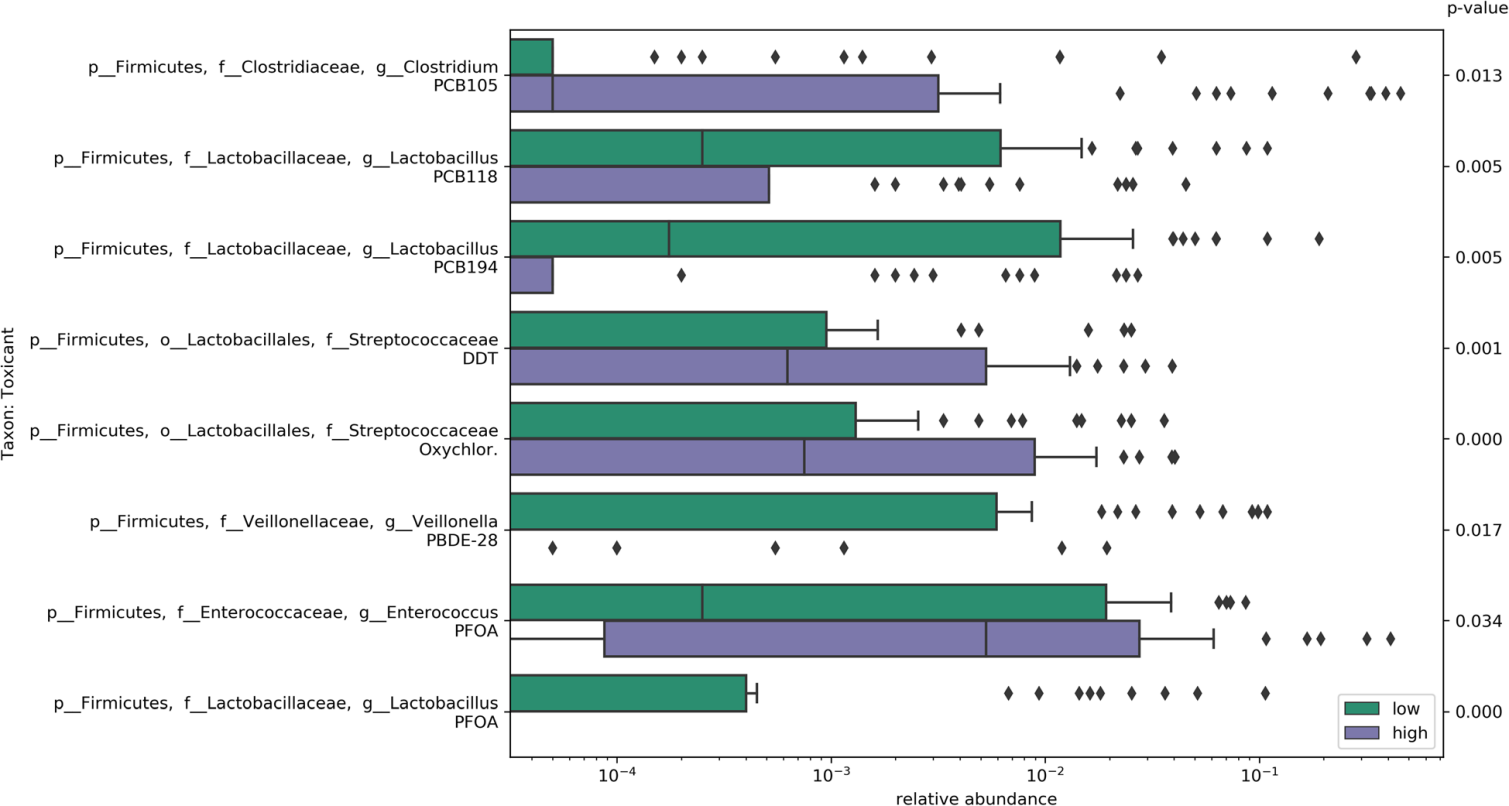
Differentially abundant sequences in the high vs. low chemical exposure groups. Restricted to exclusively breastfed infants with exposure > 80th percentile or < 20th percentile (*N* = 90), differential abundance tested using ANCOM, adjusting for gestational age. *P* values for Mann-Whitney test comparing mean log relative abundance where *p* < 0.05 after Bonferroni correction. We assigned lineages by starting from the sub-OTU tip, following the path up to the root and collecting taxonomic labels along this path. Deblurred FASTA sequences are in Additional file 1: Table S4. Taxa are differentially abundant where the proportion of rejected hypotheses within each taxon is greater than 0.7 (more conservative), except for PCB118 (0.6)

Next, we tested differential abundance of microbes in infants with relatively high vs. low exposure to individual chemicals, using analysis of composition of microbiomes (ANCOM) with a Benjamini-Hochberg correction, and adjusted for gestational age [27]. We detected differential abundance of some sub-OTUs in those exposed > 80th vs. < 20th percentile, and assigned lineages following the position of the sub-OTU sequence in the Greengenes reference tree and collecting taxonomic labels along the path to the root. Infants in the high exposure group had some differentially abundant sub-OTUs within Firmicutes, particularly the genus *Lactobacillus* (Fig. 2). Infants in the high perfluorooctanoic acid (PFOA) exposure group lacked a sub-OTU from the lineage of *Lactobacillus zeae* (*p* < 0.001) and 1.1-fold more of a sub-OTU of the genus *Enterococcus* (*p* = 0.03). Infants in the high dioxin-like PCBs exposure group had 1.8-fold lower *Lactobacillus gasseri* (*p* = 0.005) and 1.8-fold greater abundance of *Clostridium perfingens* (*p* = 0.013). High organochlorine pesticide exposure showed 0.9-fold more of a sub-OTU within the genus *Streptococcus* (*p* = 0.001), while the high PBDE-28 exposure group had 2.8-fold less *Veillonella dispar* (*p* = 0.017). The deblurred FASTA sequences are in Additional file 1: Table S3. Using a closed-reference OTU table for the same raw reads, more taxa were differentially abundant in the high breast milk-toxicant exposure group, notably PCB-167 (Additional file 1: Table S4). In addition to differential OTUs within Firmicutes, we detected lower Actinobacteria (*Bifidobacterium bifidum*, *Corynebacterium*, *Eggerthella lenta*) and Bacteroidetes (*Bacteroides fragilis* and other unidentified species of *Bacteroides*).

### Dioxin-like PCB-167 associates with relative abundance and more α-diversity of the predicted functional profile

**Fig. 3:**
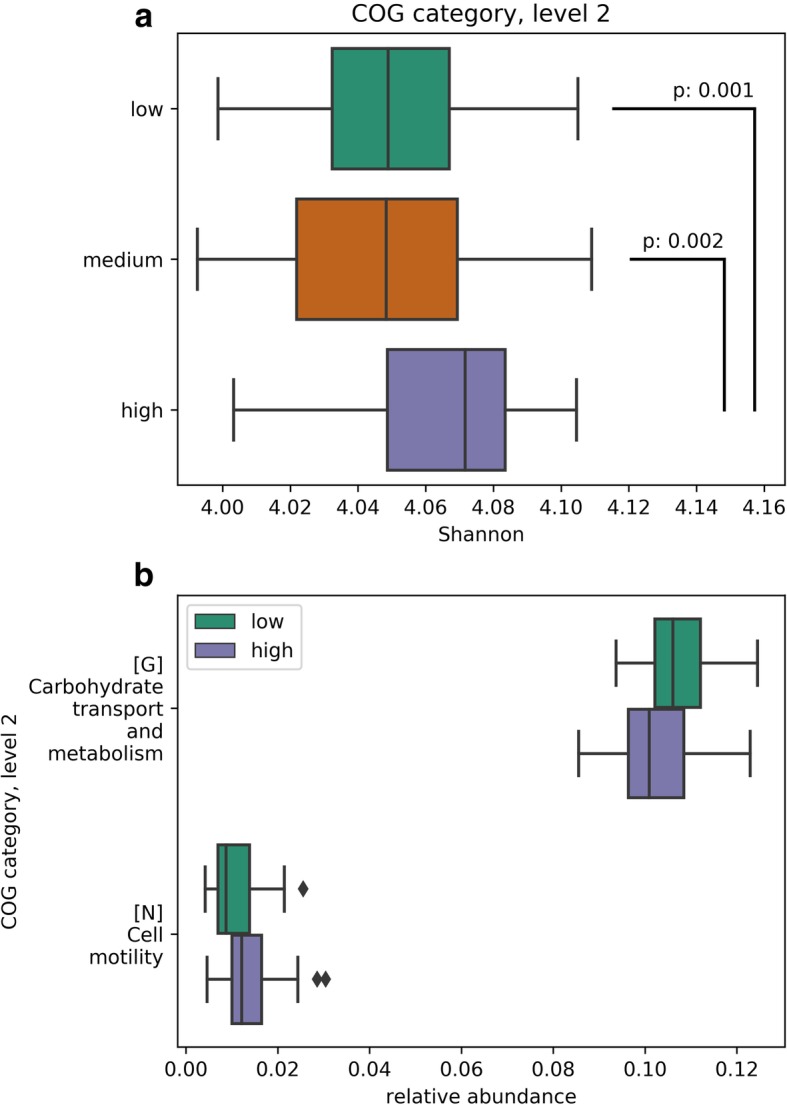
Metagenome prediction based on clusters of orthologous groups of proteins (COG) for the infant gut microbiome according to low, medium, and high breast milk PCB-167 exposure groups. These plots show the significant results from Mann-Whitney test where *p* < 0.05 after Bonferroni correction. Shannon diversity of COG features is higher in the medium and high PCB-167 exposure groups (**a**). There is differential abundance of COG pathways in the high versus low PCB-167 exposure groups (**b**)

**Fig. 4:**
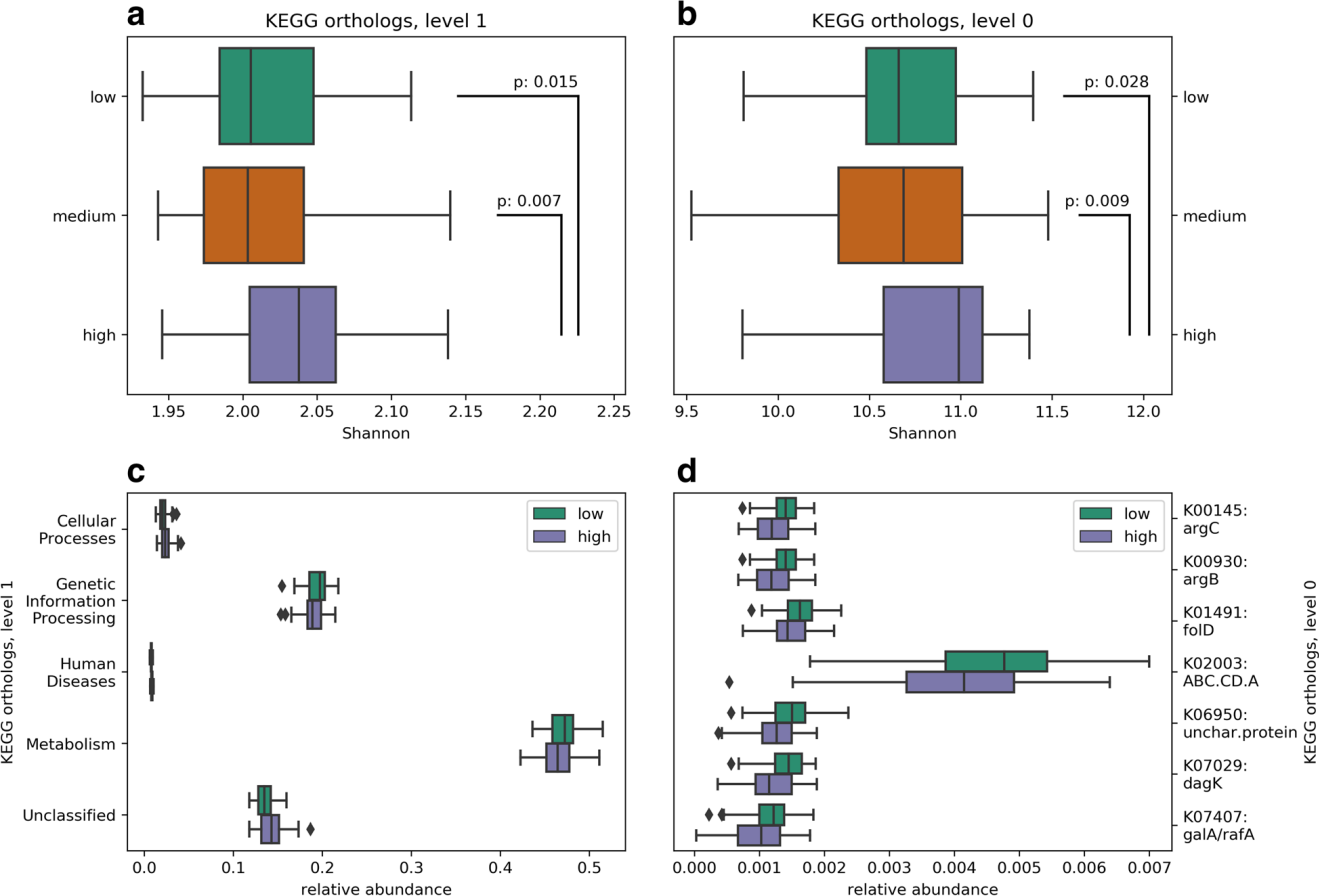
Metagenome prediction of metabolic pathways of the Kyoto Encyclopedia of Genes and Genomes (KEGG) for the infant gut microbiome according to low, medium and high breast milk PCB-167 exposure groups. These plots show the significant results from Mann-Whitney test where *p* < 0.05 after Bonferroni correction. Shannon diversity of KEGG pathways (**a**) and enzymes (**b**) is higher in the medium and high PCB-167 exposure groups. There is differential abundance of KEGG pathways (**c**) and enzymes (**d**) in the high versus low PCB-167 exposure groups

Since a taxonomic group might be replaced by another while preserving its functional contribution to the microbial community, we used PICRUSt to infer the microbiome functional profile based on clusters of orthologous groups of proteins (COG) and alternatively by players in metabolic pathways of the Kyoto Encyclopedia of Genes and Genomes (KEGG) [28]. We tested relative abundance of the various features in “high” vs. “low” exposure groups, and compared α- and β-diversity of the functional profile across exposure groups. Infants exposed to milk with higher PCB-167 had a larger functional spectrum: significantly higher Shannon diversity in COG (Fig. 3a) and KEGG (Fig. 4a). The microbiome of higher exposed infants also had 0.2-fold enrichment of “cell motility” associated predicted proteins (*p* = 0.003) and 0.05-fold less abundant “carbohydrate transport and metabolism” (*p* = 0.006) (Fig. 3b). They also had upregulated predicted proteins of “cellular processes,” “human diseases,” and “unclassified” pathways, and less abundance of predicted proteins involved in “genetic information processing” and “metabolism” pathways (Fig. 4c). Seven enzymes involved with amino acid synthesis, one carbon pool by folate, and lipid metabolism pathways were less abundant in the high PCB-167 exposure community compared with low (Fig. 4d). Other toxicants were less consistently associated with differences in the predicted metagenome (Additional file 1: Figures S4 and S5).

### Toxicants associate with lower concentrations of short-chain fatty acids, except PCB-167 and PFOA, which associate with higher concentrations

**Fig. 5:**
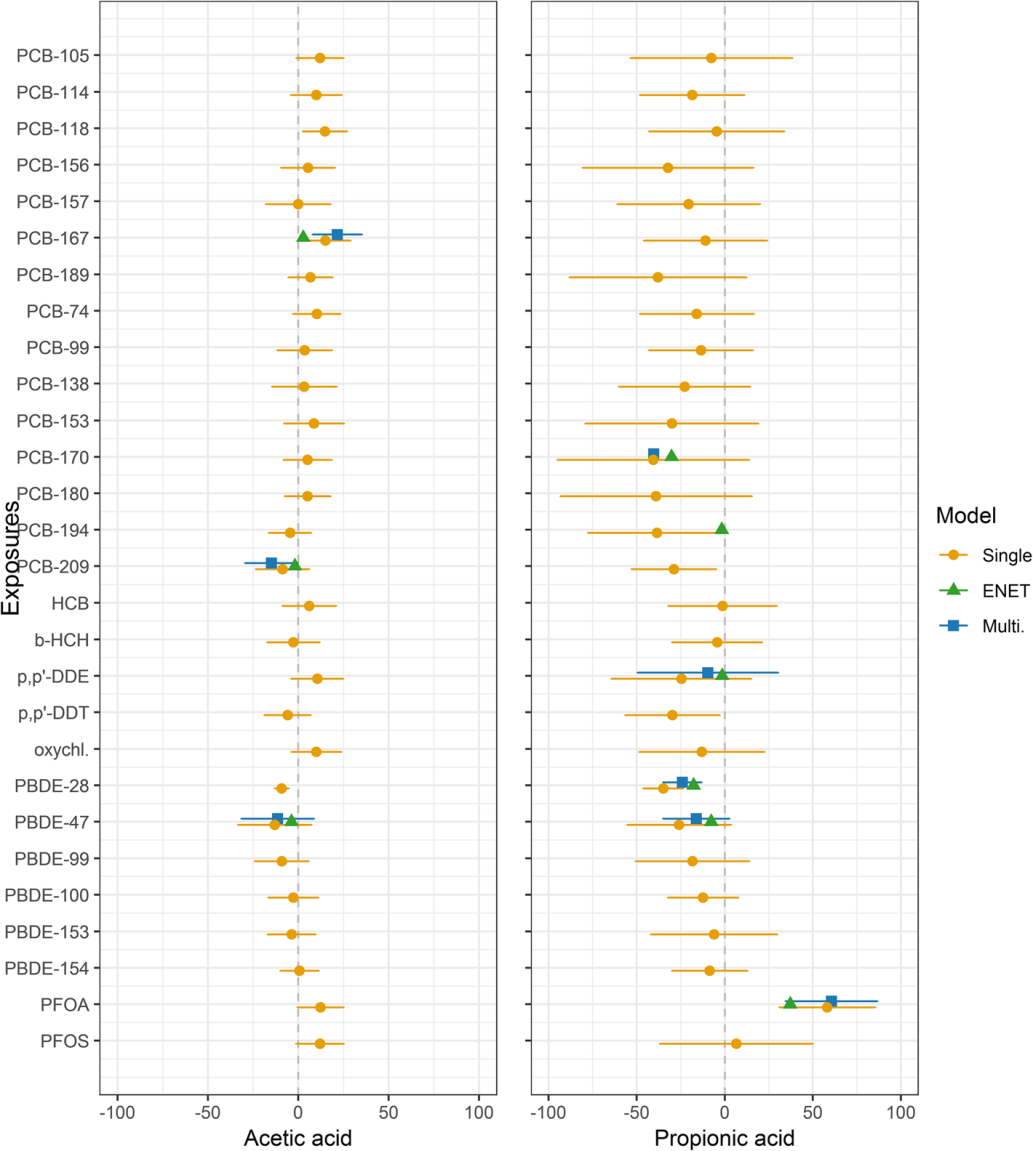
Environmental chemicals in breast milk associate with short-chain fatty acids at 1 month. Exposure units are ng/g lipid except for PFOA and PFOS (ng/L). All models adjusted for proportion of feeding from breast milk, gestational age, and C-section (yes/no). The point indicates the β estimate, the horizontal line the 95% CI, as percentage change relative to the mean of the SCFA, per 1 SD increase in exposure. ENET selected exposures (SDs in above units): *p*,*p*′-DDE (54), PCB-167 (0.7), PCB-170 (4.4), PCB-194 (1.1), PCB-209 (0.1), PBDE-28 (0.5), PBDE-47 (5.0), PFOA (34.0). ENET (green triangle) indicates chemical selected by and estimate derived from penalized elastic net using the minimum CV-MSE. Multipollutant model estimate (blue circle) from unpenalized linear regression with exposures selected via elastic net. For propionic acid, we excluded PCB-194, the exposure collinear with PCB-170 that had the lowest ENET estimate. Substitution of PCB-170 with PCB-194 did not materially affect results. Single pollutant models (orange circle) are unadjusted for other toxicants

We studied SCFAs measured in fecal samples (*n* = 70). At 1 month of age, infant fecal samples were dominated by acetic (91.7 ± 7.4%) and propionic acids (5.4 ± 5.1%) (Additional file 1: Table S2). Elastic net selected a number of toxicants as predictors of acetic and propionic acid in models adjusting for confounders (C-section, preterm delivery, maternal diversity 4 days after birth, and proportion of meals given through breastfeeding at 1 month). Multipollutant models explained 20% and 34% of variance in acetic and propionic acid, respectively, increasing to 25% and 48% when the confounders were included (Fig. 5). PCB-209 and PBDE-47 were associated with less acetic acid (− 15% [95% CI − 29% to − 0.4%] and − 11% [95% CI − 31% to 9%], respectively, statistically non-significant for the latter). Brominated flame retardants were also associated with less propionic acid (− 24% [95% 95% CI − 35% to − 13%] for PBDE-28, and − 16% [95% CI − 35% to 3%] for PBDE-47), as was PCB-170 (− 40% [95% CI − 102% to 21%], statistically non-significant for the latter two). Conversely, dioxin-like PCB-167 and PFOA were associated with more acetic acid (22%, 95% CI 8% to 35%) and propionic acid (61%, 95% CI 35% to 87%). Associations were generally imprecise for other SCFAs (low concentrations and large proportion below LOD) (Additional file 1: Figure S6).

## Discussion

Children are developmentally exposed to environmental toxicants abundant in breastmilk. At 1 month postpartum, some of these chemicals were associated with aspects of infant gut microbial composition and function. These novel findings could reveal a mode of action for persistent toxicants not previously considered; However, these findings should be interpreted with caution and require replication in other studies.

### PBDEs

PBDE-28 was associated with 4% less Shannon diversity. Additionally, infants highly exposed to PBDE-28 had samples with relatively less abundance of *Veillonella*. PBDE-28 and PBDE-47 were also associated with less propionic and acetic acids, the signaling metabolites that play an important role in immune system development [29]. We speculate that these lower levels of SCFAs may in part be explained by relatively lower abundance of *Veillonella* in the high exposed, as these bacteria are known to utilize lactate in the intestine, fermenting it to both propionic and acetic acids [30]. The potential of brominated flame retardants for disrupting microbiome composition and function has been demonstrated experimentally. In a small study, mice fed PBDE-47 or PBDE-99 had decreased microbial richness, differential abundance of some taxa, and disrupted bile acid metabolism compared with control mice [31]. The acute doses of PBDEs by oral gavage (48.5 mg/kg body weight for BDE-47), although higher than environmental exposures, were estimated to result in circulating levels at concentrations similar to that found in human populations. PBDE-71 exposure at environmentally realistic concentrations led to decreased bacterial diversity of the zebra fish gut microbiome and disrupted metabolic functions such as energy metabolism, virulence, respiration, cell division, cell signaling, and stress response [32]. However, we also note that for Shannon diversity, the differences associated with PBDE-28 were much less than that from either preterm delivery (15% less than term delivery) or any formula feeding (11% more compared to exclusive breastfeeding).

### PFASs

PFOS was associated with 5% less microbiome α-diversity. By comparison, C-section, known to disrupt the microbiome, was associated with 9% less phylogenetic diversity in this population (while preterm delivery and full formula feeding were not associated). The PFOS finding was robust to sensitivity analyses including the addition/exclusion of other potential confounders and restriction to term births. Furthermore, there was greater dissimilarity between the communities in the low and high PFOS exposure groups than within them. In an experimental setting, mice fed PFOS through oral gavage had a significant decrease in abundance of bacteria [33]. However, another study of mice with dietary exposure to PFOS at doses corresponding to general population and occupational exposure found no significant differences in gut microbial diversity relative to the control group [34]. They did observe differential abundance of bacteria within Firmicutes and Bacteroidetes, and high-dose PFOS exposure significantly induced butanoate metabolism. Here, we did not find a statistically significant association between PFOS and the SCFA metabolites. In contrast, higher PFOA exposure was associated with both more propionic acid, absence of a sub-OTU within the genus *Lactobacillus*, and greater relative abundance of a sub-OTU of *Enteroccocus*. In rodents, propionic acid enhances adipocyte differentiation of 3 T3-L1 pre-adipocytes via increased expression of GPR43 and peroxisome proliferator-activated receptor γ (PPARγ) [35], as does PFOA [36]. PFASs continue to be a concern for human health, as evidenced by the recent lowering of the European Food Safety Authority’s tolerable weekly intake [37], and the interaction between these compounds and the gut microbiome requires further investigation.

Dioxin-like PCB-167 was associated with greater β-diversity of the gut microbiome, enriched metabolic activity, and more acetic acid. This may indicate a more heterogeneous response to exposure, leading to a larger functional spectrum in more exposed communities. In experimental studies, the AhR mediates dioxin-like compounds’ toxicity and exposure increased butyrate and propionate in AhR^+/+^ but not AhR^−/−^ mice [38], while larval exposure to PCB-126 increased phylogenetic diversity in the frog gut [39]. Exposure may perturb community structure, selecting for specialized/more tolerant microbes able to degrade the chemical [40]. This could allow colonization by potential opportunistic microbes; here, infants with higher PCB-105 exposure had greater abundance of *Clostridium perfringens*. In mice, dioxin-elicited changes in the host decreased *B*. *fragilis* [41], which, in our study was relatively lower in abundance in the higher breastmilk toxicants exposure group, as were some *Lactobacillus*. Due to financial constraints, we only had measured levels of the less toxic of the dioxin-like PCBs; however, we expect these to be moderately correlated with dioxins and other more toxic dioxin-like compounds [3]. Given our findings and experimental studies, a more detailed investigation of dioxins and the human gut microbiota is warranted.

### Organochlorine pesticides

There were fewer associations with organochlorine pesticides. High oxychlordane and dichlorodiphenyltrichloroethane (*p*,*p*′-DDT) exposed communities were associated with greater relative abundance of a sub-OTU of the genus *Streptococcus*, while the metabolite dichlorodiphenyldichloroethylene (*p*,*p′*-DDE) was selected as a predictor of less propionic acid. In rats, coliform bacteria metabolize *p*,*p*′-DDT to *p*,*p*′-DDD [42], which is probably relevant for humans.

### Non-dioxin like PCBs

These compounds were associated with gut microbial function (decreased acetic and propionic acid), with less evidence for a disturbed community composition. Limited experimental evidence reports that mice orally exposed to non-dioxin-like PCBs had decreased abundance of gut bacteria, primarily Proteobacteria [43].

### Strengths and limitations

This study is based on a prospective birth cohort with rich questionnaire data to assess potential confounding. We had extensive exposure assessment of persistent chemicals at the time when fecal samples were collected, providing substantial information on the multiple breast milk toxicants to which gut bacteria were exposed. Our methods allowed for adjustment for confounding from co-occurring toxicants in linear regressions, although we could not consider the exposure profile of co-occurring toxicants when assessing low, medium, and high exposure groups of individual toxicants. PCB-167 influenced a number of microbiome metrics; however, given the high correlation with other PCBs (*r* = 0.77 – *r* = 0.92), these may represent common effects. Additionally, other unmeasured compounds may be more influential confounders (i.e., arsenic [44]). We assessed breast milk concentrations as direct exposure for the bacteria and not child blood concentrations (although they are correlated in early life [45]), which could influence gut microbiota through host physiology.

Using Deblur increases resolution, and reduces false positive annotations [24]. We detected more differentially abundant taxa using a Greengenes closed-reference table, thus some taxonomic differences may be expressed in species- to genus-level rather than sub-OTU level. This could be followed up with in vitro studies of toxicant effects on strains and species from the same genus.

We had a reasonable sample size for the α-diversity analyses; however, SCFA analyses were in 70 infants, and should be interpreted cautiously. Arsenic and diazinon perturb the gut microbiata in a sex-specific manner in mice [46, 47]; there was no interaction between toxicants and sex on α-diversity, but for SCFAs we could not test this.

All toxicant classes were associated with some alterations in composition and function, but not consistently across all metrics, or toxicants. This could be due to specific chemicals only affecting particular aspects of the microbiome, misclassification of exposure, statistical methods, or chance findings. The sensitivity and resilience of gut microbiota to environmental toxicants has been demonstrated in fish [48], and it may be difficult to detect small, transient effects in an observational design.

Preterm babies, whose gut microbiota could be more susceptible to the effect of toxicants due to immaturity of their immune system, were over-represented. In the linear regressions, adjusting for preterm delivery did not influence the toxicant effect estimates, although restricting to term births (22.5% reduction in study population) affected the interpretation of PCB-167, which became associated with decreased α-diversity.

Forty percent of mothers in the NoMIC cohort did not deliver milk, and these women were not breastfeeding (15%), or preterm (39% vs. 26% of those who delivered milk), or if they were breastfeeding exclusively breastfed for a shorter period (2.3 vs. 4.2 months), possibly indicating difficulties breastfeeding. However, there were no significant differences in infant gut diversity in the full cohort compared with our study population, so we do not expect this to bias our results.

Breast milk is an evolutionary development containing numerous specialized bioactive substances. Oligosaccharides, milk lipids, secretory IgA, and hormones are released into the milk and are uniquely adapted to the individual baby in response to the mother’s living conditions. Protection against infections and a small beneficial effect on IQ are well documented health benefits of breastfeeding [2]. Furthermore, there is a growing understanding of the role of breast milk bacteria in seeding the infant gut [49]. Here, maternal-reported formula feeding at 1 month (i.e., non-exclusive breastfeeding) was associated with more Shannon diversity in their infants (with stronger effect size than from the toxicants), and the implications of such on child health should be investigated in a targeted study. Although the toxicant concentrations were lipid-normalized, we recognize that there is a complex relation between lipophilic chemicals and fatty acids, with a possible effect on the gut microbiome. Potential interactions between lipids, bioactive substances, bacteria, and environmental toxicants in breast milk are an avenue for future research.

The milk was sampled between 2002 and 2006 from women in Norway. In World Health Organization surveys 2005–2010, breast milk from Norway had higher levels of dioxin-like PCBs (expressed as toxic equivalency factor, 3 pg TEQs/g lipid) and sum of 6 indicator PCBs (62 ng/g lipid) than the less industrialized countries of the southern hemisphere (i.e., Australia 1.8 pg TEQs/g lipid and < 20 ng/g lipid), but not among the highest (i.e., Czech Republic 7 pg TEQs/g and 380 ng/g lipid). However, regardless of level, surveyed countries had levels of dioxin-like PCBs and sum of PCBs in human milk at one to two orders of magnitude above those considered toxicologically safe in early childhood [3, 50]. By contrast, the sum of DDT in Scandinavian breast milk was the lowest (< 100 μg/kg lipid), with other European countries comparatively higher (i.e., Czech Republic 130 μg/kg lipid, and the highest in the tropical countries using DDT for vector control i.e. India > 1000 μg/kg lipid). PBDE levels are also relatively low in Norway [51]. Due to restrictions, these chemicals are in decline [52], although exposure continues through dust and food, especially in countries with lower environmental controls. These findings are relevant for the general population due to continued contamination of fish and meat.

## Conclusions

Our results suggest that environmental toxicants in breast milk, notably PBDE-28, PFOA, PFOS, and dioxin-like PCB-167, influence infant gut microbial composition and function. These novel findings must be interpreted with caution, and should be replicated in independent populations. It is unclear whether these potential toxicant-induced alterations have implications for child health, and this needs studying both in this cohort and in countries with higher contamination.

## Methods

### Study population and data collection

The Norwegian Microbiota Cohort (NoMIC) is a prospective birth cohort [21–23]. Mothers were recruited at the maternity ward of Østfold county hospital (2002–2005), two consecutive term births per preterm delivery. Fluency in Norwegian and residency in the county were inclusion criteria. Mothers were asked to collect and freeze one fecal sample from themselves at 4 days postpartum, as well as samples from their infants when they were 4, 10, 30, 120, 365, and 730 days old. Participants were asked to collect by hand a 25-ml breast milk sample each morning for eight consecutive days, between 2 weeks and 2 months postpartum [20], but minor changes in sampling protocol were also accepted. Sampling was undertaken on multiple days to reduce within-subject variability in estimated level of exposure. The milk was stored in a 250-ml container in the freezer. When the mothers had filled the container, the milk samples and fecal samples were collected by study personnel, kept frozen during transport to the Norwegian Institute of Public Health (NIPH), and stored at − 20 °C upon arrival. DNA was extracted after all samples were collected [21, 53]. Six hundred one women agreed to participate, 89% returned fecal samples, leaving a cohort of 552 children. Three hundred twenty-one mothers also delivered breastmilk samples with measured toxicants, corresponding to 333 children (including multiple births); 5 did not have microbiome information due to lost samples leaving 328 children with measurement of toxicants and gut microbiome diversity at any time point. We focused on 1 month, a sensitive period when the microbiome undergoes rapid development [54], and 87% of women were exclusively breastfeeding; whereas feces sampled at later time points could be influenced by other factors such as antibiotic use, introduction of solid food, and diet. Three hundred seven infants had both chemicals measured and fecal samples at 1 month. We excluded twins and triplets who may have different feeding patterns and thus the toxicants sampled in milk were not representative of their exposure (*n* = 26), infants whose mothers reported no breastfeeding at 1 month (*n* = 3), or infants with antibiotics use 14 days prior to fecal sampling (n = 3). Two hundred sixty-seven infants were in our α-diversity analysis (Additional file 1: Figure S7). To assess differences in microbiome composition between infants grouped by low (< 20th), medium (≥ 20th–< 80th), and high (≥ 80th percentile) breast milk toxicant exposure, we restricted to exclusively breastfed babies, since we could not adjust for confounders in those analyses (*n* = 239). SCFAs were not available for all children due to lack of sample volume, thus we studied microbiota function in a subset of participants (*n* = 70).

We obtained information on gestational age, maternal smoking, and birth weight and length through the Norwegian Medical Birth Registry, and additional important covariates, including maternal education, antibiotics use, breastfeeding, and C-section, from the 1-month questionnaire.

### Exposure variables

Mothers sampled their breast milk at a mean (SD) age of 31.4 (19.9) days using the WHO protocol [20]. Due to financial constraints, 28 chemicals were analyzed in breast milk samples in 3 laboratories: non-dioxin-like PCBs, mono-ortho dioxin-like PCBs, organochlorine pesticides, PBDEs, and PFAS (Additional file 1: Table S6). University of Life Sciences-NMBU measured PCBs and organochlorine pesticides in 15 samples using liquid-liquid extraction, gravimetrical lipid determination, and clean-up with sulfuric acid [53, 55, 56]. Following this, the laboratory at The Department of Environmental Exposure and Epidemiology, Norwegian Institute of Public Health (NIPH) established methods and analyzed the lipophilic chemicals using liquid-liquid extraction and gas chromatography–mass spectrometry (GC/MS) with negative chemical ionization [57, 58]. The majority of PFAS samples were measured in breast milk using high-performance liquid chromatography/tandem mass spectrometry (LC-MS/MS) at the NIPH [57, 59], with additional samples measured at Vrije University, Institute for Environmental Studies [60]. Measured concentrations of the lipophilic chemicals were normalized by dividing by total lipid content in the specific milk sample. We replaced values below the limit of detection (LODs) by a randomly imputed number between zero and LOD.

### Outcomes

Mothers were in close contact with health personnel and reminded to collect fecal samples at 1 month using a standard protocol.

#### Sequencing and data processing

We extracted DNA using the Earth Microbiome Project protocol: (http://press.igsb.anl.gov/earthmicrobiome/emp-standard-protocols/dna-extraction-protocol/). We sequenced 100 nt from the V4 region of the 16S rRNA gene with the Illumina HiSeq instrument. We used a recently developed sub-operational-taxonomic-unit approach, Deblur, which uses error profiles to obtain putative error-free sequences from Illumina sequencing platforms [24]. By removing noise, Deblur gives a higher resolution than OTU-based analyses or analyses of raw sequence data, and because it is reference free, it may pick up sequences of novel bacteria that are not represented in existing databases. To control for variation in sequencing coverage, the data were rarified at a depth of 20,000 sequences per sample, which lead to the removal of 8 samples. Data processing was performed in the Quantitative Insights Into Microbial Ecology (QIIME) pipeline version 1.9.1. [61]. More detailed information on DNA extraction, sequencing, and data processing is provided in the Supplemental Material.

#### Gut microbiota composition: α-diversity, β-diversity, differential abundance of taxa

We used three α-diversity measures: (i) Shannon diversity, the total number of species (species richness) weighted for their relative abundances (species evenness); (ii) Faith’s phylogenetic diversity, the amount or proportion of branch length in a phylogenetic tree that leads to different organisms (species richness); and (iii) the number of observed unique sub-OTUs. To study variation in diversity in the bacterial community in the exclusively breastfed children based on low, medium, or high toxicant concentrations, we calculated β-diversity using unweighted and weighted UniFrac [62]. In order to obtain a phylogenetic tree for diversity computation, we used Qiime2’s fragment-insertion [63] to phylogenetically place the sub-OTU sequences into the reference Greengenes 13.8 99% identity tree [64].

We also tested for differentially abundant taxa (sub-OTUs) as described below.

#### SCFAs

Two laboratories analyzed fecal samples for eight SCFAs using published analytical methods [65–67]. Briefly, distillates of fecal material were analyzed with gas chromatography and quantified using flame ionization detection. We assessed SCFAs with > 50% above LOD: acetic, propionic, *n*-butyric, *i*-butyric, and *i*-valeric acids (Additional file 1: Table S2).

### Covariates

We selected potential confounding factors a priori using directed acyclic graphs (Additional file 1: Figure S8). The minimum adjustment set to assess the effect of breast milk toxicants on gut diversity/SCFAs at 1 month was proportion of meals given through breast milk (vs. formula feeding, continuous 0–1), preterm delivery (Yes/No), maternal gut α-diversity, and C-section (Yes/No).

### Statistics

For microbiome α-diversity analyses, we imputed missing values for exposures and covariates using multiple imputation by chained equations to generate 20 imputed data sets [68, 69]. Correlations between exposures were assessed using Spearman’s rank correlation coefficients. To assess associations between breastmilk toxicants and gut microbiota α-diversity, we adopted two regression approaches, in which we standardized exposures to one SD, and adjusted for identified covariates. First, to select among individual toxicants, we used elastic net regression modeling, a hybrid penalized method robust to extreme correlations among the predictors [25, 26]. We selected α = 0.9 and optimized λ using tenfold cross-validation repeated 10 times based on minimum standard error, and unpenalized covariates [70], and repeated in each of the 20 multiply imputed datasets, considering the exposures which were selected (β ≠ 0) in more than half of the models as noteworthy [71]. We then used generalized linear models to obtain unbiased (unpenalized) estimates and assessed all pollutants individually for comparison, and then fitted multipollutant models with the elastic net-selected exposures.

We investigated group differences for low (< 20th), medium (≥ 20–≤ 80th), and high (> 80th percentile) breast milk toxicants. First, we assessed β-diversity using weighted and unweighted UniFrac, testing significance of pairwise groups with PERMANOVA [72]. Second, we investigated differences in sub-OTU abundance between low vs. high groups using the analysis of composition of microbiomes (ANCOM) framework [27]. ANCOM accounts for the compositional nature of the taxa relative abundances and is based on the analysis of difference in pairwise log-ratios of microbial OTU abundances/relative abundances, between comparison groups of interest. For each taxon, we computed a statistic indicating the number of significantly different pairwise log-ratios while controlling for false discoveries. We applied ANCOM with a Benjamini-Hochberg correction at 5% level of significance, and adjusted for gestational age. For comparison with other studies, we assigned lineages to the identified differentially abundant sub-OTUs. Instead of using machine learning approaches like classifying against the RDP, we used the phylogenetic tree produced for diversity computation and its assigned Greengenes taxonomy labels to obtain lineages for the sub-OTUs: For every sub-OTU sequence, we started from the inserted sub-OTU tip and followed the path up to the root while collecting taxonomic labels along this path. Third, we analyzed the predicted metagenome. We tested for differentially abundant functions using a discrete false-discovery rate correction [73]. For the same relative abundances, we computed α-diversity of the normalized relative PICRUSt abundances by applying the Shannon metric and used two-sided Mann-Whitney tests to check for significant differences between the three exposure groups. We then computed β-diversity distances for the same relative abundances via the Bray-Curtis metric and performed PERMANOVA tests with 9999 permutations to check for statistically significant differences within vs. between the groups of “high,” “medium,” and “low” labeled samples. We used Bonferroni correction for multiple hypothesis testing with *p* < 0.05 to consider two groups as different.

Finally, we tested the relation between toxicants and SCFAs, using elastic net regression and generalized linear models, with a natural logarithm to transform the SCFAs and adjusting for confounders. We did not include maternal gut diversity in the SCFA analyses, as there were 56% missing and the multiple imputation models for the SCFAs would not converge. All regression models were tested for and met the assumptions of normality, homoscedasticity, and linearity.

We used STATA 14.0 for multiple imputation and generalized linear regression and R programme version 3.2 [74] for ANCOM and elastic net (using the glmnet package [25]). We used scikit-bio 0.5.1 for PERMANOVA tests.

#### Sensitivity of regression analyses

We tested the sensitivity of regression model estimates: restricting to complete case, breast milk sample collection age < 60 days, exclusive breastfeeding, term births, ln-transformed exposures, and excluding extreme values. We tested the inclusion/exclusion of: household pets, infant antibiotics in the first 2 weeks of life (as those with antibiotics in the 2 weeks prior to sampling were excluded from the analyses), parity, smoking at the start of pregnancy, maternal BMI, and education. We tested interactions between the elastic net-selected compounds sex, maternal BMI and preterm birth for the α-diversity models.

## Additional files

Additional files 1: Supplemental material **Table S1.** Characteristics of NoMIC current study population, full cohort and general population of birth-giving mothers in Norway. **Table S2.** Distribution of diversity measures and short-chain fatty acids in infant fecal samples at 1 month. **Table S3.** Greengenes lineage for deblurred FASTA sequences. **Table S4.** Differentially abundant taxa in the high (>80th percentile) vs. low (<20th percentile) chemical exposure groups based on Greengenes 13.1 closed-reference OTU Table (97% identity). **Table S5.** Association between individual toxicants and Shannon diversity, phylogenetic diversity and observed sub-OTUs restricted to term births (≥37 weeks gestational age) (*n* = 207) . **Figure S1.** Spearman’s correlations between concentrations of environmental chemicals in breast milk at 1 month. **Figure S2.** Characterization of the gut microbiota samples of infants at 1 month. **Figure S3.** Environmental chemicals in breastmilk associated with infant gut microbiome α-diversity at 1 month. **Figure S4.** Metagenome prediction based on Clusters of Orthologous Groups of proteins (COG) for the infant gut microbiome according to low, medium and high breast milk dioxin-like PCB exposure groups. **Figure S5**. Metagenome prediction of metabolic pathways of the Kyoto Encyclopedia of Genes and Genomes (KEGG) for the infant gut microbiome according to low, medium and high breast milk chemical exposure groups. **Figure S6.** Environmental chemicals in breast milk associated with short-chain fatty acids at 1 month. **Figure S7.** Flowchart of participants in NoMIC study. **Figure S8.** Direct acyclic graph of the relation between toxicants in breast milk and infant gut diversity. **Methods.** Additional information on extraction, sequencing and data processing. (DOCX 1150 kb)

Additional file 2: Sample R script and Stata syntax for unadjusted analyses. (ZIP 8 kb)

Additional file 3: Data files. (ZIP 65 kb)

## Abbreviations

ANCOM: Analysis of composition of microbiomes
ENET: Elastic-net
HCB: Hexachlorobenzene
LOD: Limit of detection
*p*,*p*′*-*DDE: Dichlorodiphenyldichloroethylene
*p*,*p*′*-*DDT: Dichlorodiphenyltrichloroethane
PBDE: Polybrominated diphenyl ethers
PCB: Polychlorinated biphenyl
PERMANOVA: Permutational multivariate analysis of variance
PFOA: Perfluorooctanoic acid
PFOS: Perfluorooctanesulfonic acid
POP: Persistent organic pollutant
QIIME: Quantitative Insights Into Microbial Ecology
β-HCH: Beta-hexachlorocyclohexane

## References

[1] Koleva PT (2015). Microbial programming of health and disease starts during fetal life. Birth Defects Res C Embryo Today.

[2] Victora CG (2016). Breastfeeding in the 21st century: epidemiology, mechanisms, and lifelong effect. Lancet.

[3] van den Berg M (2017). WHO/UNEP global surveys of PCDDs, PCDFs, PCBs and DDTs in human milk and benefit-risk evaluation of breastfeeding. Arch Toxicol.

[4] UNEP (2001). Stockholm Convention on Persistent Organic Pollutants.

[5] UNEP (2009). Stockholm Convention on Persistent Organic Pollutants.

[6] Mogensen UB (2015). Breastfeeding as an exposure pathway for perfluorinated alkylates. Environmental Science & Technology.

[7] Lehmann GM (2018). Environmental chemicals in breast milk and formula: exposure and risk assessment implications.

[8] Schug TT (2013). PPTOX III: environmental stressors in the developmental origins of disease—evidence and mechanisms. Toxicol Sci.

[9] Vrijheid M (2016). Environmental pollutants and child health—a review of recent concerns. Int J Hyg Environ Health.

[10] Jin Y (2017). Effects of environmental pollutants on gut microbiota. Environ Pollut.

[11] Claus SP, Guillou H, Ellero-Simatos S (2016). The gut microbiota: a major player in the toxicity of environmental pollutants?. Npj Biofilms And Microbiomes.

[12] Gensollen T (2016). How colonization by microbiota in early life shapes the immune system. Science.

[13] Kaplan JL, Shi HN, Walker WA (2011). The role of microbes in developmental immunologic programming. Pediatr Res.

[14] Wang M, Monaco MH, Donovan SM (2016). Impact of early gut microbiota on immune and metabolic development and function. Semin Fetal Neonatal Med.

[15] Rooks MG, Garrett WS (2016). Gut microbiota, metabolites and host immunity. Nat Rev Immunol.

[16] Oleskin AV, Shenderov BA (2016). Neuromodulatory effects and targets of the SCFAs and gasotransmitters produced by the human symbiotic microbiota. Microb Ecol Health Dis.

[17] Flint HJ (2012). The role of the gut microbiota in nutrition and health. Nat Rev Gastroenterol Hepatol.

[18] Heindel JJ, Newbold R, Schug TT (2015). Endocrine disruptors and obesity. Nat Rev Endocrinol.

[19] Rossignol DA, Genuis SJ, Frye RE (2014). Environmental toxicants and autism spectrum disorders: a systematic review. Transl Psychiatry.

[20] WHO (2007). Fourth WHO-coordinated survey of human milk for persistent organic pollutants in cooperation with UNEP -guidelines for developing a national protocol.

[21] Eggesbø M (2011). Development of gut microbiota in infants not exposed to medical interventions. APMIS.

[22] Rudi K (2007). Alignment-independent comparisons of human gastrointestinal tract microbial communities in a multidimensional 16S rRNA gene evolutionary space. Appl Environ Microbiol.

[23] Mandal S (2016). Fat and vitamin intakes during pregnancy have stronger relations with a pro-inflammatory maternal microbiota than does carbohydrate intake. Microbiome.

[24] Amir A, et al. Deblur rapidly resolves single-nucleotide community sequence patterns. mSystems. 2017;2(2) 10.1128/mSystems.00191-16 PMC5340863 28289731

[25] Friedman J, Hastie T, Tibshirani R (2010). Regularization paths for generalized linear models via coordinate descent. J Stat Softw.

[26] Zou H, Hastie T (2005). Regularization and variable selection via the elastic net. Journal of the Royal Statistical Society: Series B (Statistical Methodology).

[27] Mandal S (2015). Analysis of composition of microbiomes: a novel method for studying microbial composition.

[28] Langille MGI (2013). Predictive functional profiling of microbial communities using 16S rRNA marker gene sequences. Nat Biotechnol.

[29] Kabat AM, Srinivasan N, Maloy KJ (2014). Modulation of immune development and function by intestinal microbiota. Trends Immunol.

[30] Kumari M, Kozyrskyj AL (2017). Gut microbial metabolism defines host metabolism: an emerging perspective in obesity and allergic inflammation. Obes Rev.

[31] Li, C.Y., et al., PBDEs altered gut microbiome and bile acid homeostasis in male C57BL/6 mice. 2018. 46(8): p. 1226–1240. 10.1124/dmd.118.081547 PMC6053593 29769268

[32] Chen L (2018). Acute exposure to PBDEs at an environmentally realistic concentration causes abrupt changes in the gut microbiota and host health of zebrafish. Environ Pollut.

[33] Xu C (2017). Estrogen receptor beta mediates hepatotoxicity induced by perfluorooctane sulfonate in mouse. Environ Sci Pollut Res.

[34] Lai KP (2018). Dietary exposure to the environmental chemical, PFOS on the diversity of gut microbiota, associated with the development of metabolic syndrome. Front Microbiol.

[35] Canfora EE, Jocken JW, Blaak EE (2015). Short-chain fatty acids in control of body weight and insulin sensitivity. Nat Rev Endocrinol.

[36] Yamamoto J (2015). Perfluorooctanoic acid binds to peroxisome proliferator-activated receptor γ and promotes adipocyte differentiation in 3T3-L1 adipocytes. Bioscience, Biotechnology, and Biochemistry.

[37] Chain, E.P.o.C.i.t.F (2018). Risk to human health related to the presence of perfluorooctane sulfonic acid and perfluorooctanoic acid in food.

[38] Zhang L (2015). Persistent organic pollutants modify gut microbiota-host metabolic homeostasis in mice through aryl hydrocarbon receptor activation. Environ Health Perspect.

[39] Kohl KD (2015). Larval exposure to polychlorinated biphenyl 126 (PCB-126) causes persistent alteration of the amphibian gut microbiota. Environ Toxicol Chem.

[40] Yan T, LaPara TM, Novak PJ (2006). The reductive dechlorination of 2,3,4,5-tetrachlorobiphenyl in three different sediment cultures: evidence for the involvement of phylogenetically similar Dehalococcoides-like bacterial populations. FEMS Microbiol Ecol.

[41] Stedtfeld RD (2017). Modulatory influence of segmented filamentous bacteria on transcriptomic response of gnotobiotic mice exposed to TCDD. Front Microbiol.

[42] Mendel JL, Walton MS (1966). Conversion of p,p-DDT to p,p-DDD by intestinal Flora of the rat. Science.

[43] Choi JJ (2013). Exercise attenuates PCB-induced changes in the mouse gut microbiome. Environ Health Perspect.

[44] Lu K (2014). Arsenic exposure perturbs the gut microbiome and its metabolic profile in mice: an integrated metagenomics and metabolomics analysis. Environ Health Perspect.

[45] Needham LL (2011). Partition of environmental chemicals between maternal and fetal blood and tissues. Environmental Science & Technology.

[46] Chi L (2016). Sex-specific effects of arsenic exposure on the trajectory and function of the gut microbiome. Chem Res Toxicol.

[47] Gao B (2017). Sex-specific effects of organophosphate Diazinon on the gut microbiome and its metabolic functions. Environ Health Perspect.

[48] Narrowe AB (2015). Perturbation and restoration of the fathead minnow gut microbiome after low-level triclosan exposure. Microbiome.

[49] Pannaraj PS (2017). Association between breast milk bacterial communities and establishment and development of the infant gut microbiome. JAMA Pediatr.

[50] Van den Berg M (2006). The 2005 World Health Organization reevaluation of human and mammalian toxic equivalency factors for dioxins and dioxin-like compounds. Toxicol Sci.

[51] Frederiksen M (2009). Human internal and external exposure to PBDEs—a review of levels and sources. Int J Hyg Environ Health.

[52] Nost TH (2017). The impacts of emission trends of POPs on human concentration dynamics: lessons learned from a longitudinal study in Norway (1979-2007). Int J Hyg Environ Health.

[53] Eggesbø M (2009). Levels of hexachlorobenzene (HCB) in breast milk in relation to birth weight in a Norwegian cohort. Environ Res.

[54] Morelli L (2008). Postnatal development of intestinal microflora as influenced by infant nutrition. J Nutr.

[55] Eggesbø M (2011). Associations between brominated flame retardants in human milk and thyroid-stimulating hormone (TSH) in neonates. Environ Res.

[56] Polder A (2009). Levels of chlorinated pesticides and polychlorinated biphenyls in Norwegian breast milk (2002-2006), and factors that may predict the level of contamination. Sci Total Environ.

[57] Thomsen C (2010). Changes in concentrations of perfluorinated compounds, polybrominated diphenyl ethers, and polychlorinated biphenyls in Norwegian breast-milk during twelve months of lactation. Environmental Science & Technology.

[58] Thomsen C, Liane VH, Becher G (2007). Automated solid-phase extraction for the determination of polybrominated diphenyl ethers and polychlorinated biphenyls in serum—application on archived Norwegian samples from 1977 to 2003. J Chromatogr B Analyt Technol Biomed Life Sci.

[59] Haug LS, Thomsen C, Becher G (2009). A sensitive method for determination of a broad range of perfluorinated compounds in serum suitable for large-scale human biomonitoring. J Chromatogr A.

[60] Forns J (2015). Perfluoroalkyl substances measured in breast milk and child neuropsychological development in a Norwegian birth cohort study. Environ Int.

[61] Kuczynski J, et al. Using QIIME to analyze 16S rRNA gene sequences from microbial communities, in current protocols in microbiology: Wiley; 2005. 10.1002/9780471729259.mc01e05s27 PMC4477843 23184592

[62] Lozupone C, Knight R (2005). UniFrac: a new phylogenetic method for comparing microbial communities. Appl Environ Microbiol.

[63] Janssen S, et al. Phylogenetic placement of exact amplicon sequences improves associations with clinical information. 2018;3(3):e00021–18. 10.1128/mSystems.00021-18 PMC5904434 29719869

[64] McDonald D (2012). An improved Greengenes taxonomy with explicit ranks for ecological and evolutionary analyses of bacteria and archaea. The ISME journal.

[65] Zijlstra JB (1977). Pretreatment methods prior to gaschromatographic analysis of volatile fatty acids from faecal samples. Clin Chim Acta.

[66] Midtvedt A-C (1988). Development of five metabolic activities associated with the intestinal microflora of healthy infants. J Pediatr Gastroenterol Nutr.

[67] Hoverstad T (1984). Short-chain fatty acids in the normal human feces. Scand J Gastroenterol.

[68] Rubin DB (1987). Multiple imputation for nonresponse in surveys.

[69] van Buuren S (2007). Multiple imputation of discrete and continuous data by fully conditional specification. Stat Methods Med Res.

[70] Hastie T, Tibshirani R, Friedman J. The elements of statistical learning: data mining, inference, and prediction. 2nd ed: Springer-Verlag; 2009.

[71] Wood AM, White IR, Royston P (2008). How should variable selection be performed with multiply imputed data?. Stat Med.

[72] Anderson MJ (2001). A new method for non-parametric multivariate analysis of variance. Austral Ecology.

[73] Jiang L (2017). Discrete false-discovery rate improves identification of differentially abundant microbes. mSystems.

[74] R Core Team (2013). R: A Language and environment for statistical computing.

