# Supplementary material for: Environmental toxicants in breast milk of Norwegian mothers and gut bacteria composition and metabolites in their infants at 1 month
Source: Microbiome. 2019 Feb 27;7:34. doi: 10.1186/s40168-019-0645-2 (PMC6393990; doi:10.1186/s40168-019-0645-2)
Supplement: Supplementary file 1 — Table S1. Characteristics of NoMIC current study population, full cohort and general population of birth-giving mothers in Norway. Table S2. Distribution of diversity measures and short-chain fatty acids in infant fecal samples at 1 month. Table S3. Greengenes lineage for deblurred FASTA sequences. Table S4. Differentially abundant taxa in the high (>80th percentile) vs. low (<20th percentile) chemical exposure groups based on Greengenes 13.1 closed-reference OTU Table (97% identity). Table S5. Association between individual toxicants and Shannon diversity, phylogenetic diversity and observed sub-OTUs restricted to term births (≥37 weeks gestational age) (n = 207) . Figure S1. Spearman’s correlations between concentrations of environmental chemicals in breast milk at 1 month. Figure S2. Characterization of the gut microbiota samples of infants at 1 month. Figure S3. Environmental chemicals in breastmilk associated with infant gut microbiome α-diversity at 1 month. Figure S4. Metagenome prediction based on Clusters of Orthologous Groups of proteins (COG) for the infant gut microbiome according to low, medium and high breast milk dioxin-like PCB exposure groups. Figure S5. Metagenome prediction of metabolic pathways of the Kyoto Encyclopedia of Genes and Genomes (KEGG) for the infant gut microbiome according to low, medium and high breast milk chemical exposure groups. Figure S6. Environmental chemicals in breast milk associated with short-chain fatty acids at 1 month. Figure S7. Flowchart of participants in NoMIC study. Figure S8. Direct acyclic graph of the relation between toxicants in breast milk and infant gut diversity. Methods. Additional information on extraction, sequencing and data processing. (DOCX 1150 kb) [file 40168_2019_645_MOESM1_ESM.docx]

**Supplemental Material**

**Environmental toxicants in breast milk of Norwegian mothers and gut bacteria composition and metabolites in their infants at 1 month**

Nina Iszatt, Stefan Janssen, Virissa Lenters, Cecilie Dahl, Hein Stigum, Rob Knight, Siddhartha Mandal, Shyamal Peddada, Antonio González-Peña, Tore Midtvedt, Merete Eggesbø.

Contents

[Table S1: Characteristics of NoMIC current study population, full cohort and general population of birth-giving mothers in Norway 2](#_Toc865542)

[Table S2: Distribution of diversity measures and short-chain fatty acids in infant fecal samples at 1 month. 3](#_Toc865543)

[Table S3. Greengenes lineage for deblurred FASTA sequences. 4](#_Toc865544)

[Table S4: Differentially abundant taxa in the high (>80th percentile) vs. low (<20th percentile) chemical exposure groups based on Greengenes 13.1 closed-reference OTU table (97 % identity). 5](#_Toc865545)

[Table S5: Association between individual toxicants and Shannon diversity, phylogenetic diversity and observed sub-OTUs restricted to term births (≥37 weeks gestational age) (n=207) 6](#_Toc865546)

[Figure S1: Spearman’s correlations between concentrations of environmental chemicals in breast milk at 1 month. Chemical concentrations untransformed (ng/g lipid, except PFOA and PFOS, ng/L). 7](#_Toc865547)

[Figure S2: Characterization of the gut microbiota samples of infants at 1 month. 8](#_Toc865548)

[Figure S3: Environmental chemicals in breastmilk associated with infant gut microbiome 9](#_Toc865549)

[Figure S4: Metagenome prediction based on Clusters of Orthologous Groups of proteins (COG) for the infant gut microbiome according to low, medium and high breast milk dioxin-like PCB exposure groups. 10](#_Toc865550)

[Figure S5: Metagenome prediction of metabolic pathways of the Kyoto Encyclopedia of Genes and Genomes (KEGG) for the infant gut microbiome according to low, medium and high breast milk chemical exposure groups. 10](#_Toc865551)

[Figure S6: Environmental chemicals in breast milk associated with short-chain fatty acids at 1 month. 11](#_Toc865552)

[Figure S7: Flowchart of participants in NoMIC study 12](#_Toc865553)

[Figure S8: Direct acyclic graph of the relation between toxicants in breast milk and infant gut diversity 13](#_Toc865554)

[Methods: Additional information on extraction, sequencing and data processing 14](#_Toc865555)

[References 14](#_Toc865556)

Table S1: Characteristics of NoMIC current study population, full cohort and general population of birth-giving mothers in Norway

| Characteristic | NoMIC  study population 1 | | NoMIC  study population 2 | | NoMIC  study population 3 | | NoMIC  full cohort | General population^a^ |
| --- | --- | --- | --- | --- | --- | --- | --- | --- |
|  | n=267^b^ | Mean (SD) or % | n=239 | Mean (SD) or % | n=70 | Mean (SD) or % | n=608 | n=126,182 |
| *Maternal factors* |  |  |  |  |  |  |  |  |
| Maternal alpha diversity |  |  |  |  |  |  |  |  |
| Shannon diversity | 113 | 4.8 (0.6) | 99 | 4.8 (0.7) | 31 | 4.8 (0.5) | 4.7 |  |
| Phylogenetic diversity | 113 | 20.6 (5.2) | 99 | 20.4 (5.3) | 31 | 20.6 (5.0) | 20.3 |  |
| sOTUs | 113 | 191.5 (60.3) | 99 | 189.0 (62.0) | 31 | 190.2 (59.6) | 187.7 |  |
| Age (y) | 267 | 30.4 (4.4) | 239 | 30.1 (4.3) | 70 | 30.8 (4.7) | 29.9 | 29 |
| Pre-pregnancy BMI (kg/m^2^) | 264 | 24.3 (4.5) | 236 | 24.1 (4.4) | 68 | 23.8 (4.3) | 24.4 |  |
| Primiparous | 267 |  | 239 |  | 70 |  |  |  |
| Yes |  | 40.8 |  | 40.6 |  | 42.9 | 44.5 | 43.9 |
| Smoking at pregnancy start | 210 |  | 186 |  | 52 |  |  |  |
| Yes |  | 10.0 |  | 9.1 |  | 5.8 | 15.8 | 13.2 |
| Education (y) | 261 |  | 234 |  |  |  |  |  |
| <12 |  | 9.2 |  | 8.5 |  | 1.4 | 12.9 |  |
| 12 |  | 18.0 |  | 16.7 |  | 20.3 | 22.2 |  |
| >12 |  | 72.8 |  | 74.8 |  | 78.3 | 64.9 |  |
| Ethnicity | 240 |  | 214 |  | 62 |  |  |  |
| Caucasian |  | 99.6 |  | 99.5 |  | 100 | 99.1 |  |
| Any maternal antibiotics during pregnancy and C-section | 260 |  | 234 |  | 68 |  |  |  |
| None |  | 55.8 |  | 55.1 |  | 60.3 | 48.2 |  |
| C-section, no antibiotics |  | 14.6 |  | 14.5 |  | 11.8 | 17.1 |  |
| Antibiotics, no C-section |  | 17.7 |  | 17.9 |  | 20.6 | 19.8 |  |
| Antibiotics and C-section |  | 11.9 |  | 12.4 |  | 7.4 | 14.9 |  |
| *Child factors* |  |  |  |  |  |  |  |  |
| Child alpha diversity | 267 |  |  |  |  |  |  |  |
| Shannon diversity |  | 2.2 (0.6) |  |  |  |  | 2.2 |  |
| Phylogenetic diversity |  | 6.3 (1.8) |  |  |  |  | 6.5 |  |
| sOTUs |  | 33.7 (15.0) |  |  |  |  | 36.3 |  |
| Birth weight, g | 267 | 3340 (785.3) | 239 | 3338 (796.6) | 70 | 3358 (856.4) | 3340^e^ | 3570 |
|  |  |  |  |  |  |  |  |  |
| Sex | 267 |  | 239 |  | 70 |  |  |  |
| Female |  | 43.5 |  | 43.1 |  | 42.9 | 47.8 | 48.8 |
| Household pets in infancy | 214 |  | 190 |  | 65 |  |  |  |
| Yes |  | 41.6 |  | 43.7 |  | 35.4 | 47.3 |  |
| Antibiotics in first 14 days postpartum | 262 |  | 235 |  | 69 |  |  |  |
| Yes |  | 11.1 |  | 11.9 |  | 11.6 | 13.5 |  |
| *Mother-child shared factors* |  |  |  |  |  |  |  |  |
| Gestational age (days) | 267 | 273 (21.8) | 239 | 272 (22.4) | 70 | 273 (24.49) | 277^e^ | 282 |
| Preterm delivery | 267 |  | 239 |  | 70 |  |  |  |
| Yes |  | 22.5 |  | 23.0 |  | 18.6 | 28.8 | 5 |
| Exclusive breastfeeding at 1 month | 267 |  | 239 |  | 70 |  |  |  |
| Yes |  | 86.5 |  | 100 |  | 84.3 | 87.0 |  |
| Milk sampling (days from birth) | 173 | 31.4 (19.9) | 163 | 37.3 (32.5) | 42 | 26.3 (16.7) |  |  |

Continuous measures described by mean (standard deviation, SD); categorical measures described by frequencies, %.

Study population 1: Alpha diversity analyses

Study population 2: Exclusively breastfed babies,

Study population 3: SCFA analyses

^a^ Available information on the general population of recent mothers was obtained through the Norwegian Medical Birth Registry (which records all births in Norway). Mothers who had given birth between 2001 and 2003 were selected and their characteristics compared to the NoMIC participants.
^b^ Number with information on described characteristic

^e^ Due to the oversampling of preterm babies, NoMIC subjects have babies with lower birth weight and gestational age.

For the N=267 study population, the following variables had missing values (%): maternal alpha diversity (58), pre-pregnancy BMI (1), primiparous (1), smoking (21), education (2), ethnicity (10), maternal antibiotics/C-section (3), household pets (20), child antibiotics first 14 days (2), breastmilk sample collection time (35).

Table S2: Distribution of diversity measures and short-chain fatty acids in infant fecal samples at 1 month.

|  | Outcome | N | % missing | % <LOD* | Mean (SD) | min | p25 | p50 | p75 | max |
| --- | --- | --- | --- | --- | --- | --- | --- | --- | --- | --- |
| Alpha diversity | Shannon | 267 | 0 | 0 | 2.23 (0.62) | 0.57 | 1.84 | 2.29 | 2.68 | 3.92 |
|  | Phylogenetic | 267 | 0 | 0 | 6.27 (1.81) | 2.9 | 5.01 | 6.03 | 7.25 | 15.54 |
|  | Observed OTUs | 267 | 0 | 0 | 33.72 (15.03) | 14.6 | 23.2 | 30.3 | 40.2 | 121.5 |
| Short-chain fatty acids (mmol/kg) | Acetic acid | 70 | 73.8 | 0 | 67.11 (37.58) | 12.18 | 40.75 | 60.11 | 90.74 | 219.56 |
|  | Propionic acid | 70 | 73.8 | 4.3 | 4.09 (4.68) | 0.06 | 1.34 | 2.26 | 5.26 | 18.95 |
|  | *n*-Butyric acid | 70 | 73.8 | 4.3 | 1.65 (3.78) | 0.02 | 0.26 | 0.53 | 1.54 | 28.54 |
|  | *i*-Butyric acid | 70 | 73.8 | 45.7 | 0.42 (0.37) | 0.04 | 0.11 | 0.34 | 0.66 | 1.51 |
|  | *i*-Valeric acid | 70 | 73.8 | 37.1 | 0.28 (0.41) | 0.03 | 0.06 | 0.12 | 0.28 | 1.79 |
|  | *n*-Valeric acid | 70 | 73.8 | 81.4 | 0.31 (0.59) | 0.02 | 0.09 | 0.13 | 0.29 | 2.24 |
|  | *n*-Caproic acid | 70 | 73.8 | 84.3 | 0.13 (0.08) | 0.04 | 0.06 | 0.13 | 0.19 | 0.32 |
|  | *i*-Caproic acid | 70 | 73.8 | 92.9 | 0.09 (0.03) | 0.06 | 0.07 | 0.09 | 0.09 | 0.13 |

* We imputed SCFAs values below LOD (<0.06 mmol/kg).

Table S3. Greengenes lineage for deblurred FASTA sequences.

| Deblurred FASTA sequence | Greengenes lineage | | |
| --- | --- | --- | --- |
|  | Family | Genus | Species |
| TACGTAGGTGGCGAGCGTTATCCGGATTTACTGGGCGTAAAGGGAGCGTAGGCGGATGATTAAGTGGGATGTGAAATACCCGGGCTCAACTTGGGTGCTG | Clostridiaceae | *Clostridium* | *perfingens* |
| TACGTAGGTGGCAAGCGTTGTCCGGATTTATTGGGCGTAAAGCGAGTGCAGGCGGTTCAATAAGTCTGATGTGAAAGCCTTCGGCTCAACCGGAGAATTG | Lactobacillaceae | *Lactobacillus* | *gasseri* |
| TACGTAGGTCCCGAGCGTTGTCCGGATTTATTGGGCGTAAAGCGAGCGCAGGCGGTTAGATAAGTCTGAAGTTAAAGGCTGTGGCTTAACCATAGTACGC | Streptococcaceae | *Streptococcus* |  |
| TACGTAGGTGGCAAGCGTTGTCCGGAATTATTGGGCGTAAAGCGCGCGCAGGCGGATAGGTCAGTCTGTCTTAAAAGTTCGGGGCTTAACCCCGTGATGG | Veillonellaceae | *Veillonella* | dispar |
| TACGTAGGTGGCAAGCGTTGTCCGGATTTATTGGGCGTAAAGCGAGCGCAGGCGGTTTCTTAAGTCTGATGTGAAAGCCCCCGGCTCAACCGGGGAGGGT | Enterococcaceae | *Enterococcus* |  |
| TACGTAGGTGGCAAGCGTTATCCGGATTTATTGGGCGTAAAGCGAGCGCAGGCGGTTTTTTAAGTCTGATGTGAAAGCCCTCGGCTTAACCGAGGAAGTG | Lactobacillaceae | *Lactobacillus* | zeae |

For identified differential sub-OTUs, we assigned lineages by starting from the sub-OTU tip of the insertion tree obtained by phylogenetically placing sub-OTU sequences into the Greengenes 13.8 reference tree and following the path up to the root and collecting taxonomic labels along this path.

Table S4: Differentially abundant taxa in the high (>80th percentile) vs. low (<20th percentile) chemical exposure groups based on Greengenes 13.1 closed-reference OTU table (97 % identity).

|  | Chemical | Green genes ID | Phylum | Class | Family | Genus | Species | High toxicant | Effect size^a^ |
| --- | --- | --- | --- | --- | --- | --- | --- | --- | --- |
| dl-PCBs | PCB-105 | 4428313 | Firmicutes | Bacilli | *Lactobacillaceae* | *Lactobacillus* |  | 🡫*** | -0.61 |
|  |  | 4451477 | Firmicutes | Clostridia | *Clostridiaceae* | *Clostridium* | *perfringens* | 🡩** | 0.03 |
|  |  | 539647 | Firmicutes | Bacilli | *Lactobacillaceae;* | *Lactobacillus* |  | 🡫* | -0.01 |
|  |  | 4479397 | Bacteroidetes | Bacteroidia | *Bacteroidaceae* | *Bacteroides* | *fragilis* | 🡫* | -0.29 |
|  | PCB-118 | 4428313 | Firmicutes | Bacilli | *Lactobacillaceae* | *Lactobacillus* |  | 🡫*** | -0.57 |
|  |  | 539647 | Firmicutes | Bacilli | *Lactobacillaceae* | *Lactobacillus* |  | 🡫** | -0.01 |
|  | PCB-156 | 4428313 | Firmicutes | Bacilli | *Lactobacillaceae* | *Lactobacillus* |  | 🡫** | -0.24 |
|  |  | 4468234 | Bacteroidetes | Bacteroidia | *Bacteroidaceae* | *Bacteroides* |  | 🡫** | -0.08 |
|  |  | 539647 | Firmicutes | Bacilli | *Lactobacillaceae* | *Lactobacillus* |  | 🡫* | -0.01 |
|  | PCB-167 | 365385 | Actinobacteria | Actinobacteria | *Bifidobacteriaceae* | *Bifidobacterium* | *bifidum* | 🡫** | -1.41 |
|  |  | 4428313 | Firmicutes | Bacilli | *Lactobacillaceae* | *Lactobacillus* |  | 🡫** | -0.18 |
|  |  | 4479397 | Bacteroidetes | Bacteroidia | *Bacteroidaceae* | *Bacteroides* | *fragilis* | 🡫** | -0.065 |
|  |  | 4457872 | Bacteroidetes | Bacteroidia | *Bacteroidaceae* | *Bacteroides* |  | 🡫** | 0 |
|  |  | 4468234 | Bacteroidetes | Bacteroidia | *Bacteroidaceae* | *Bacteroides* |  | 🡫* | -0.217 |
|  |  | 4469576 | Firmicutes | Clostridia | *Lachnospiraceae* |  |  | 🡫* | 0 |
| ndl-PCBs | PCB-99 | 941632 | Actinobacteria | Actinobacteria | *Corynebacteriaceae* | *Corynebacterium* |  | 🡫** | -0.12 |
|  | PCB-170 | 4428313 | Firmicutes | Bacilli | *Lactobacillaceae* | *Lactobacillus* |  | 🡫*** | -0.14 |
|  |  | 539647 | Firmicutes | Bacilli | *Lactobacillaceae* | *Lactobacillus* |  | 🡫** | -0.01 |
|  |  | 841635 | Proteobacteria | Betaproteobacteria | *Sutterellaceae* | *Sutterella* |  | 🡫* | 0 |
|  | PCB-180 | 4428313 | Firmicutes | Bacilli | *Lactobacillaceae* | *Lactobacillus* |  | 🡫** | -0.14 |
|  |  | 539647 | Firmicutes | Bacilli | *Lactobacillaceae* | *Lactobacillus* |  | 🡫** | -0.01 |
|  |  | 841635 | Proteobacteria | Betaproteobacteria | *Sutterellaceae* | *Sutterella* |  | 🡫* | 0 |
|  | PCB-194 | 4428313 | Firmicutes | Bacilli | *Lactobacillaceae* | *Lactobacillus* |  | 🡫*** | -0.58 |
|  |  | 539647 | Firmicutes | Bacilli | *Lactobacillaceae* | *Lactobacillus* |  | 🡫*** | -0.02 |
|  |  | 4468234 | Bacteroidetes | Bacteroidia | *Bacteroidaceae* | *Bacteroides* |  | 🡫** | -0.30 |
|  |  | 4454531 | Proteobacteria | Gammaproteobacteria | *Enterobacteriaceae* |  |  | 🡫* | -0.60 |
|  | PCB-209 | 4468234 | Bacteroidetes | Bacteroidia | *Bacteroidaceae* | *Bacteroides* |  | 🡫** | -0.16 |
|  |  | 109382 | Proteobacteria | Betaproteobacteria | *Burkholderiaceae* |  |  | 🡫* | -1.11 |
| OC pest. | HCB | 4469576 | Firmicutes | Clostridia | *Lachnospiraceae* |  |  | 🡫* | 0 |
|  | DDE | 4393532 | Actinobacteria | Coriobacteriia | *Eggerthellaceae* | *Eggerthella* | *lenta* | 🡫** | -0.01 |
|  | Oxychl. | 4428313 | Firmicutes | Bacilli | *Lactobacillaceae* | *Lactobacillus* |  | 🡫* | -0.02 |
| PBDEs | PBDE-28 | 4475758 | Firmicutes | Clostridia | *Veillonellaceae* | *Veillonella* | *dispar* | 🡫*** | -0.05 |
|  | PBDE-100 | 4347159 | Actinobacteria | Actinobacteria | *Bifidobacteriaceae* | *Bifidobacterium* | *adolescentis* | 🡩* | 0.36 |
|  |  | 824876 | Actinobacteria | Actinobacteria | *Bifidobacteriaceae* | *Bifidobacterium* |  | 🡩* | 0.06 |

Restricted to exclusively breastfed infants with exposure >80^th^ percentile or <20^th^ percentile (N=90), differential abundance tested using ANCOM, adjusting for gestational age. *Differentially abundant where the proportion of rejected hypotheses within each taxon is greater than 0.7 ** Differentially abundant taxa where the proportion of rejected hypotheses within each taxon is greater than 0.8 *** Differentially abundant taxa where the proportion of rejected hypotheses within each taxon is greater than 0.9. ^a^ ANCOM does not give effect sizes, so we calculated the difference between median log relative abundance between high vs. low exposure groups (unadjusted for gestational age).

Table S5: Association between individual toxicants and Shannon diversity, phylogenetic diversity and observed sub-OTUs restricted to term births (≥37 weeks gestational age) (n=207)

|  | Shannon Diversity | |  | Phylogenetic Diversity | |  | Observed sub-OTUs | |
| --- | --- | --- | --- | --- | --- | --- | --- | --- |
| Exposure | Single-pollutant  β (95% CI) | ENET β |  | Single-pollutant  β (95% CI) | ENET β |  | Single-pollutant  β (95% CI) | ENET β |
| PCB-105 | -0.15 (-0.25, -0.06) | -0.047 |  | -0.47 (-0.85, -0.10) | -0.029 |  | -3.62 (-6.64, -0.60) | 0 |
| PCB-114 | -0.07 (-0.17, 0.02) | 0 |  | -0.38 (-0.68, -0.08) | 0 |  | -3.06 (-5.27, -0.85) | 0 |
| PCB-118 | -0.13 (-0.23, -0.03) | 0 |  | -0.50 (-0.91, -0.10) | 0 |  | -4.00 (-7.11, -0.89) | 0 |
| PCB-156 | -0.07 (-0.17, 0.02) | 0 |  | -0.27 (-0.57, 0.04) | 0 |  | -2.37 (-4.70, -0.04) | 0 |
| PCB-157 | -0.03 (-0.13, 0.07) | 0 |  | -0.19 (-0.45, 0.06) | 0 |  | -1.75 (-3.76, 0.27) | 0 |
| PCB-167 | -0.11 (-0.21, -0.01) | 0 |  | -0.53 (-0.91, -0.14) | -0.108 |  | -4.28 (-7.16, -1.39) | -1.049 |
| PCB-189 | -0.11 (-0.22, -0.00) | 0 |  | -0.32 (-0.79, 0.14) | 0 |  | -2.78 (-6.16, 0.61) | 0 |
| PCB-74 | -0.08 (-0.16, 0.01) | 0 |  | -0.29 (-0.60, 0.03) | 0 |  | -2.36 (-4.78, 0.07) | 0 |
| PCB-99 | -0.07 (-0.16, 0.02) | 0 |  | -0.24 (-0.55, 0.07) | 0 |  | -2.08 (-4.55, 0.38) | 0 |
| PCB-138 | -0.08 (-0.19, 0.03) | 0 |  | -0.24 (-0.61, 0.12) | 0 |  | -2.18 (-4.94, 0.59) | 0 |
| PCB-153 | -0.10 (-0.22, 0.02) | 0 |  | -0.28 (-0.72, 0.15) | 0 |  | -2.78 (-6.07, 0.51) | 0 |
| PCB-170 | -0.10 (-0.20, 0.002) | 0 |  | -0.24 (-0.65, 0.18) | 0 |  | -2.31 (-5.36, 0.74) | 0 |
| PCB-180 | -0.10 (-0.21, 0.01) | 0 |  | -0.25 (-0.72, 0.21) | 0 |  | -2.56 (-6.01, 0.89) | 0 |
| PCB-194 | -0.08 (-0.18, 0.02) | 0 |  | -0.13 (-0.61, 0.36) | 0 |  | -1.43 (-4.98, 2.13) | 0 |
| PCB-209 | -0.04 (-0.13, 0.06) | 0 |  | -0.00 (-0.31, 0.31) | 0 |  | -0.48 (-2.86, 1.90) | 0 |
| HCB | -0.02 (-0.10, 0.05) | 0 |  | -0.12 (-0.44, 0.19) | 0 |  | -1.11 (-3.49, 1.28) | 0 |
| β-HCH | 0.03 (-0.04, 0.09) | 0 |  | -0.13 (-0.38, 0.13) | 0 |  | -1.16 (-2.90, 0.58) | 0 |
| *p'p’*-DDE | -0.06 (-0.16, 0.04) | 0 |  | -0.12 (-0.30, 0.05) | 0 |  | -1.09 (-2.37, 0.19) | 0 |
| *p'p’*-DDT | -0.05 (-0.15, 0.05) | 0 |  | -0.16 (-0.34, 0.02) | 0 |  | -1.06 (-2.35, 0.23) | 0 |
| Oxychlor. | -0.03 (-0.11, 0.05) | 0 |  | -0.15 (-0.44, 0.14) | 0 |  | -1.50 (-4.00, 1.01) | 0 |
| PBDE-28 | -0.08 (-0.14, -0.03) | -0.021 |  | -0.10 (-0.19, 0.00) | 0 |  | -1.16 (-1.81, -0.51) | 0 |
| PBDE-47 | -0.09 (-0.13, -0.05) | 0 |  | 0.05 (-0.15, 0.26) | 0 |  | 0.14 (-1.55, 1.82) | 0 |
| PBDE-99 | -0.08 (-0.11, -0.04) | 0 |  | 0.04 (-0.14, 0.21) | 0 |  | 0.04 (-1.36, 1.45) | 0 |
| PBDE-100 | -0.09 (-0.16, -0.01) | 0 |  | 0.05 (-0.18, 0.29) | 0 |  | 0.13 (-1.72, 1.98) | 0 |
| PBDE-153 | -0.08 (-0.19, 0.02) | 0 |  | 0.04 (-0.26, 0.34) | 0 |  | -0.55 (-2.58, 1.47) | 0 |
| PBDE-154 | -0.02 (-0.08, 0.04) | 0 |  | 0.18 (0.07, 0.29) | 0 |  | 1.06 (0.09, 2.03) | 0 |
| PFOA | 0.02 (-0.07, 0.11) | 0 |  | -0.04 (-0.33, 0.24) | 0 |  | -0.41 (-2.55, 1.72) | 0 |
| PFOS | -0.05 (-0.15, 0.05) | 0 |  | -0.33 (-0.63, -0.04) | -0.044 |  | -2.61 (-5.01, -0.20) | -0.300 |

Exposure units are ng/g lipid except for PFOA and PFOS (ng/L). All models adjusted for proportion of feeding from breast milk, maternal gut α-diversity and C-section (Yes/No). ENET selected exposures (SDs in above units): PBDE-28 (0.5), PFOS (63). ENET β indicates chemical selected by and estimate derived from penalised elastic net using the minimum CV-MSE. The single-pollutant β is unadjusted for other contaminants.


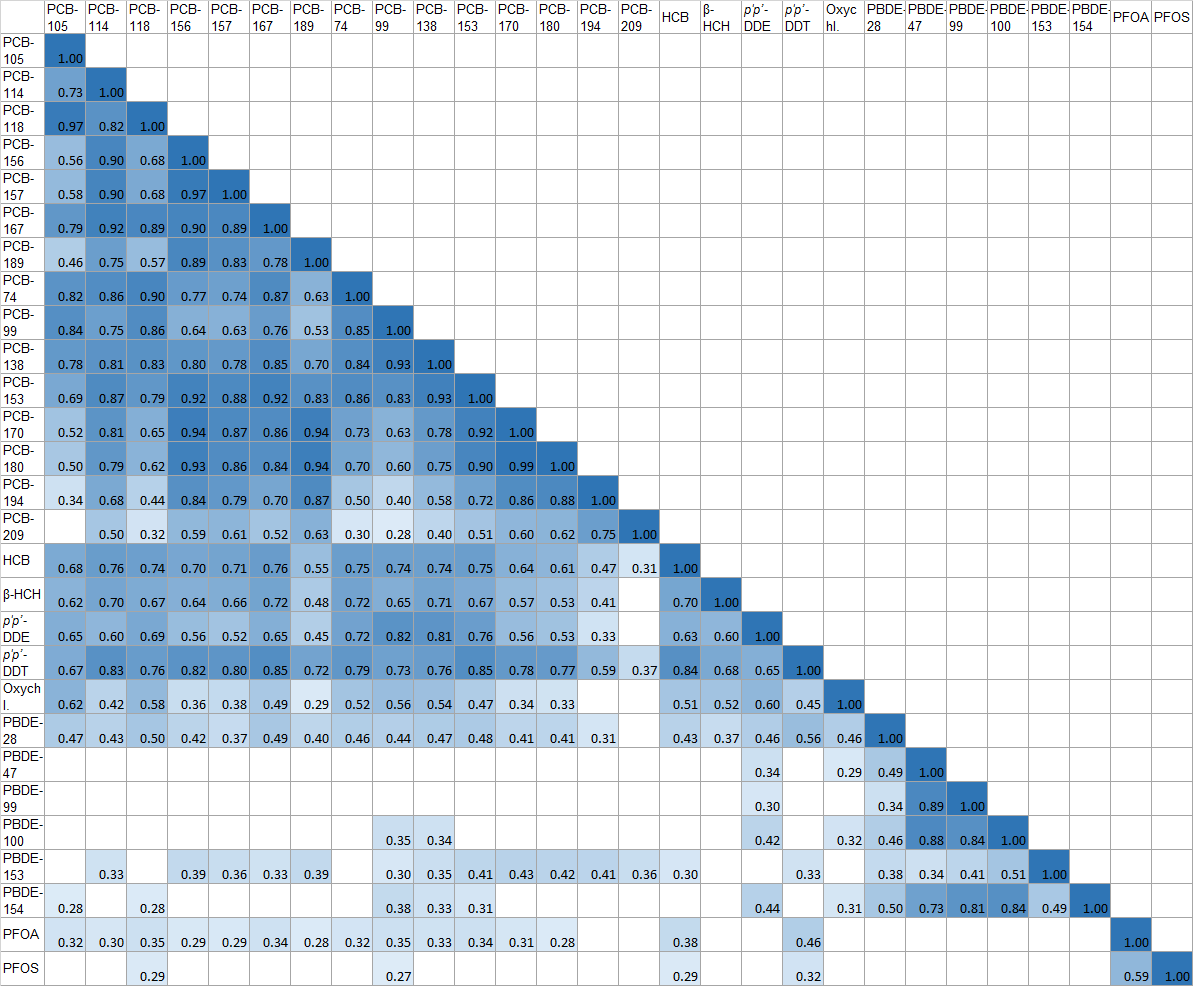


# Figure S1: Spearman’s correlations between concentrations of environmental chemicals in breast milk at 1 month. Chemical concentrations untransformed (ng/g lipid, except PFOA and PFOS, ng/L).


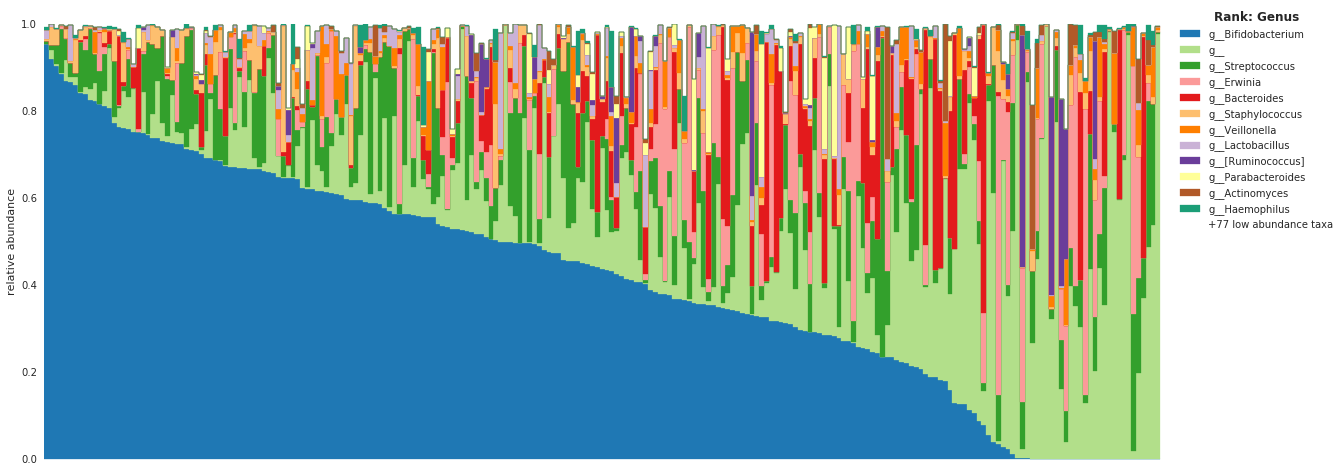


# Figure S2: Characterization of the gut microbiota samples of infants at 1 month.

Mean relative abundance of the most abundant genera in infant gut microbiota samples at one month. For identified differential sub-OTUs, we assigned lineages by starting from the sub-OTU tip of the insertion tree obtained by phylogenetically placing sub-OTU sequences into the Greengenes 13.8 reference tree and following the path up to the root and collect taxonomic labels along this path. “g_” represents all of the sub-OTUs for which we could not assign a genus.


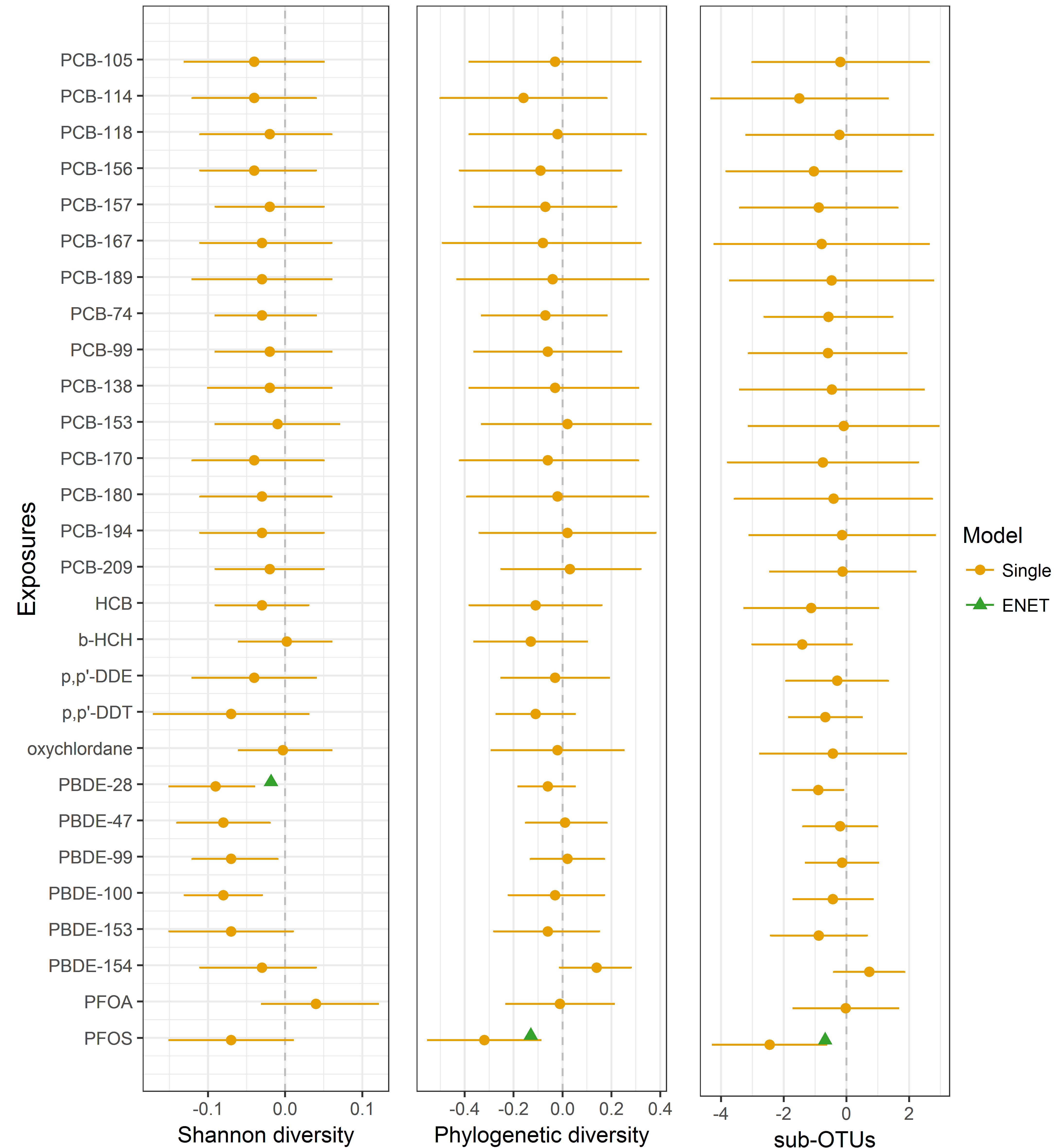


Figure S3: Environmental chemicals in breastmilk associated with infant gut microbiome **α-diversity** at 1 month. Exposure units are ng/g lipid except for PFOA and PFOS (ng/L). All models adjusted for proportion of feeding from breast milk, gestational age, maternal gut α-diversity and C-section (Yes/No). The point indicates β estimate, the horizontal line the 95 % CI, change per 1 SD increase in exposure. ENET selected exposures (SDs in above units): PBDE-28 (0.5), PFOS (63). ENET (green triangle) indicates chemical selected by and estimate derived from penalised elastic net using the minimum CV-MSE. The single pollutant model (orange circle) is unadjusted for other contaminants.


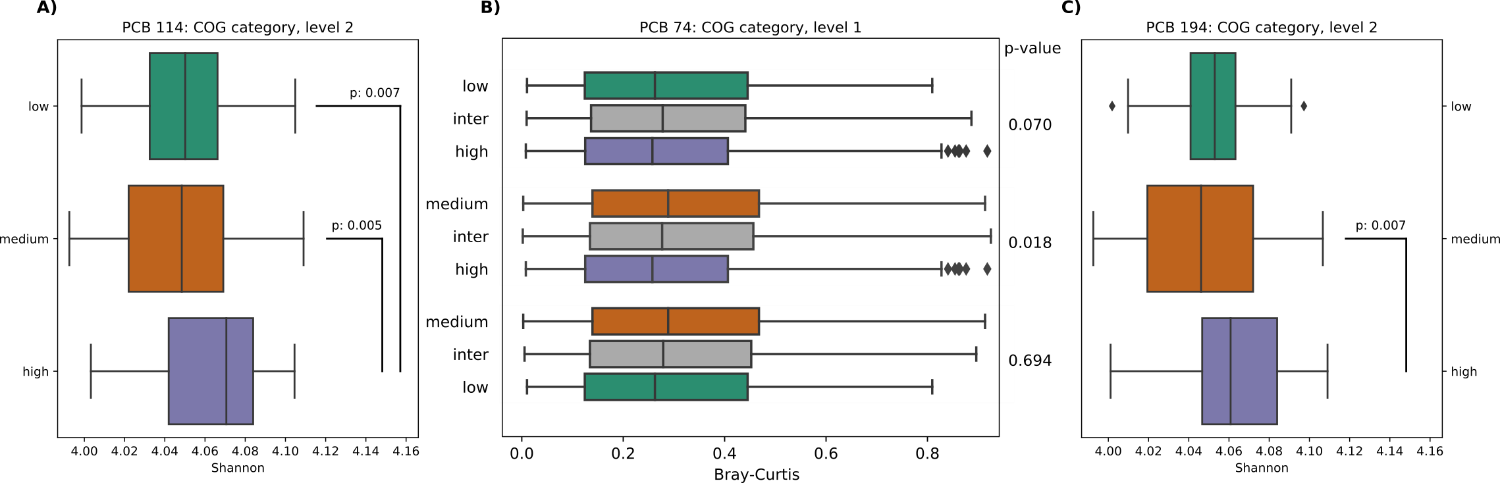


Figure S4: Metagenome prediction based on Clusters of Orthologous Groups of proteins (COG) for the infant gut microbiome according to low, medium and high breast milk dioxin-like PCB exposure groups. Shannon plots A) and C) display the significant results from Mann-Whitney test where *p*<0.05 after Bonferroni correction. Bray-Curtis plot B) results from pairwise PERMANOVA tests.

**
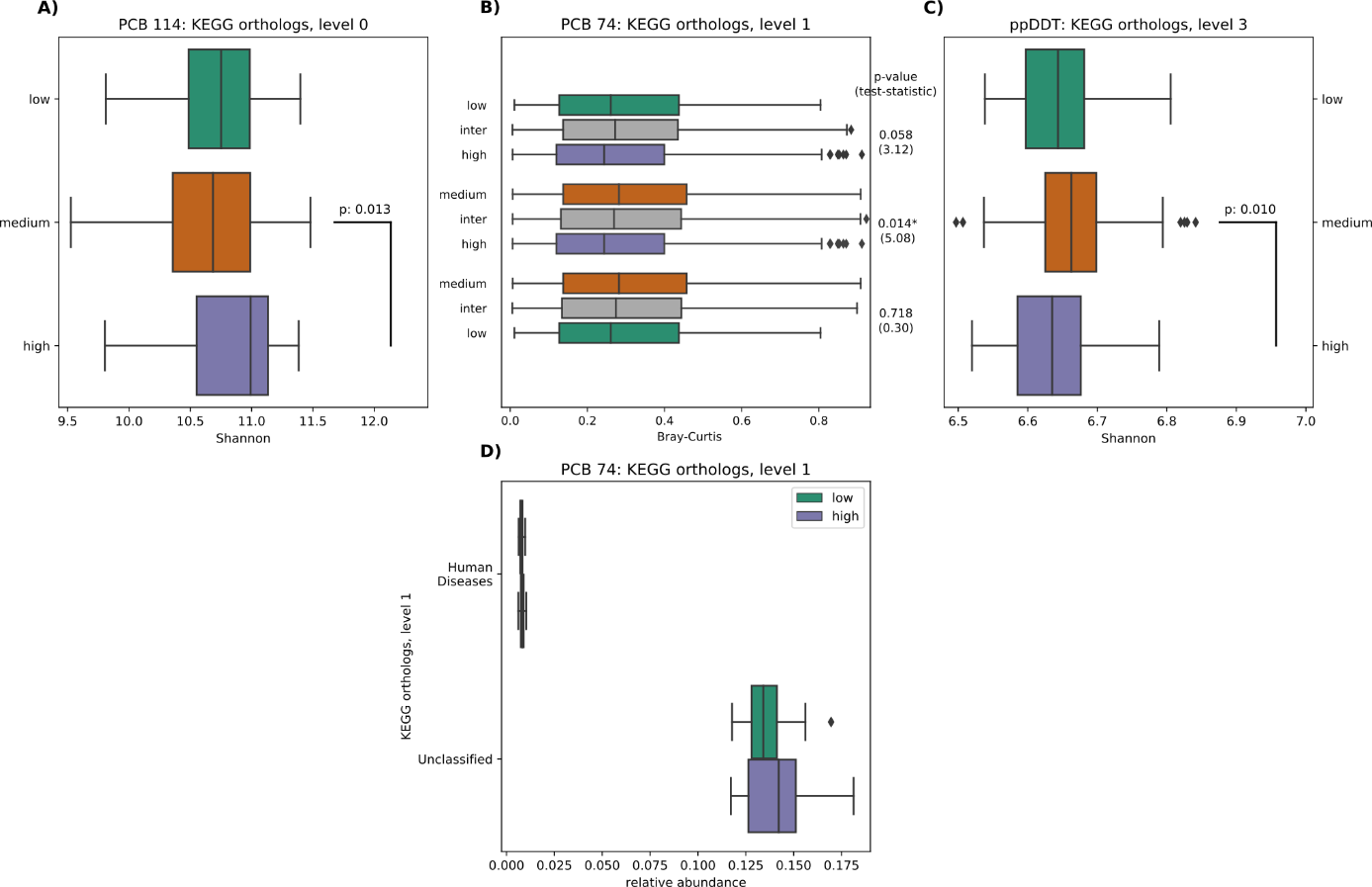
**

Figure S5: Metagenome prediction of metabolic pathways of the Kyoto Encyclopedia of Genes and Genomes (KEGG) for the infant gut microbiome according to low, medium and high breast milk chemical exposure groups. Plots A-C show the significant results from Mann-Whitney test where *p*<0.05 after Bonferroni correction. PCB-74 also showed the same pattern as in B) for KEGG orthologs levels 2 and 3 (data not shown).

**
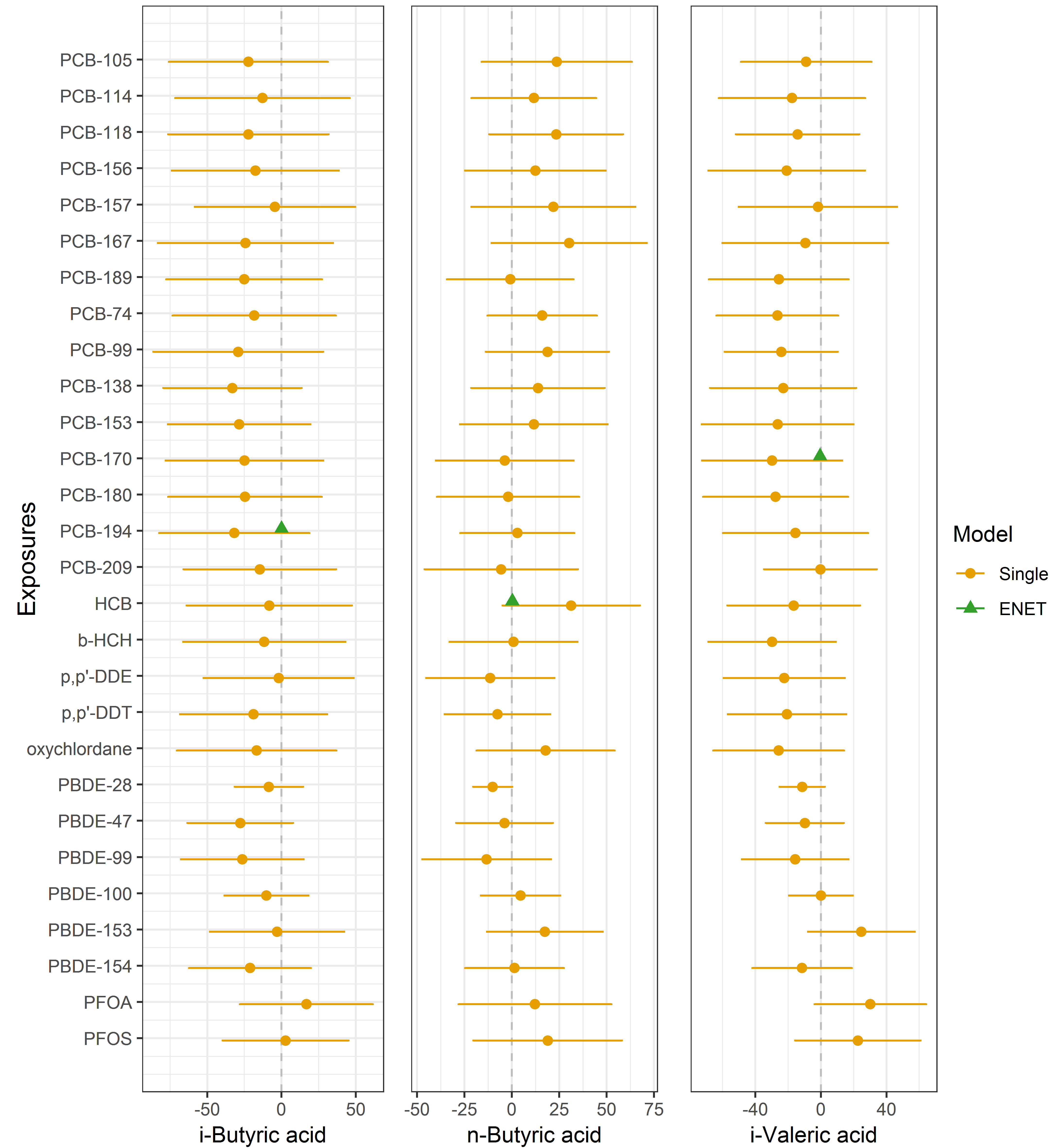
**

Figure S6: Environmental chemicals in breast milk associated with short-chain fatty acids at 1 month. Exposure units are ng/g lipid except for PFOA and PFOS (ng/L). All models adjusted for proportion of feeding from breast milk, gestational age, and C-section (Yes/No). The point indicates the β estimate, the horizontal line the 95 % CI, as percentage change relative to the mean of the SCFA per 1 SD increase in exposure. ENET selected exposures (SDs in above units): PCB-170 (4.4), PCB194 (1.1), HCB (5.0). ENET (green triangle) indicates chemical selected by and estimate derived from penalised elastic net using the minimum CV-MSE. The single pollutant model (orange circle) is unadjusted for other contaminants.

**
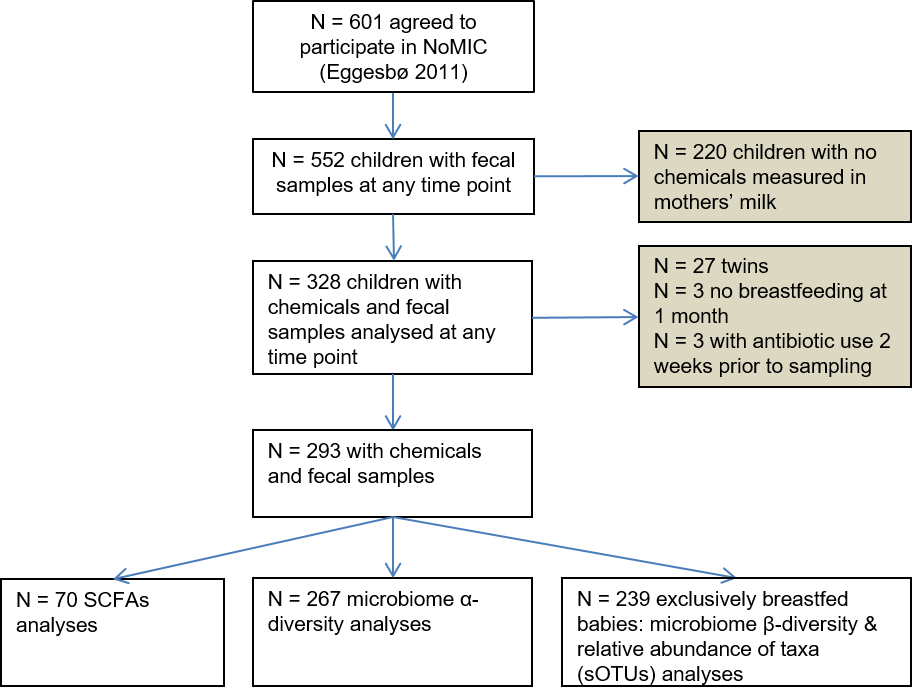
**

# Figure S7: Flowchart of participants in NoMIC study


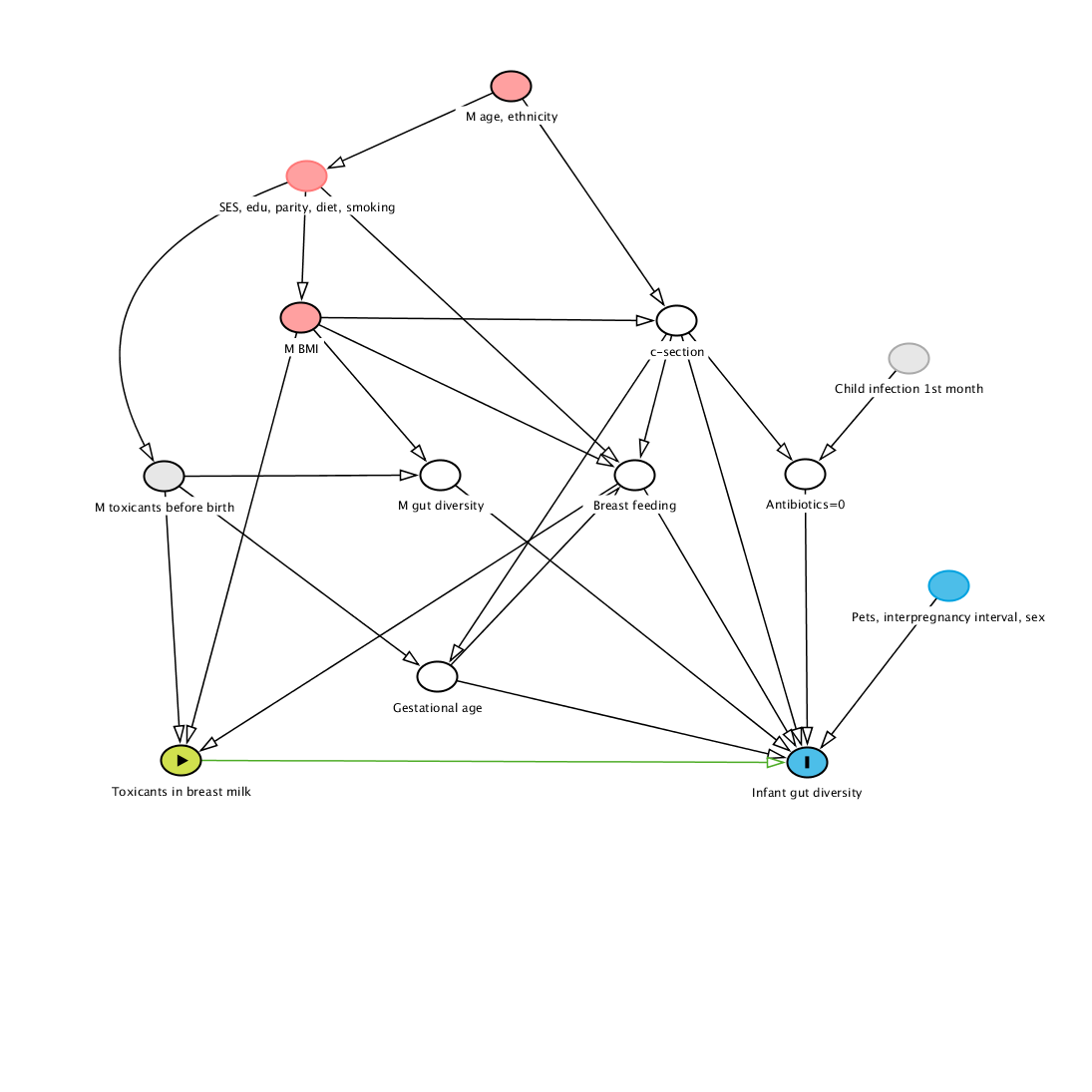


# Figure S8: Direct acyclic graph of the relation between toxicants in breast milk and infant gut diversity

Exposure

Outcome (I) or ancestor of outcome

Ancestor of both outcome and exposure

Adjusted variable

Unmeasured variable

A pink line denotes a biasing pathway. Since all the lines are black, the adjustment selection should remove bias from confounding.

# Methods: Additional information on extraction, sequencing and data processing

*Automated DNA purification of fecal samples*

Feces were prepared for analysis by adding one milliliter Solution 1 (50 mM glucose, 25 mM Tris–HCl pH 8.0, and 10 mM EDTA pH 8.0) per 0.2 g faeces. The samples were mixed by vortexing and left for 30-60 min. on ice before 400 µl of the supernatant was diluted 1:2 in 4 M guanidinium thiocyanate (GTC). Five hundred microliters of sample were transferred to a sterile FastPrep®-tube (Qbiogene Inc., Carlsbad, CA, USA) containing 250 mg glass beads (106 microns and finer, Sigma-Aldrich, Steinheim, Germany), and samples were homogenized for 40 seconds in FastPrep® Instrument (Qbiogene). Wells in a 96-well Greiner U-plate (Greiner bio-one, Frickenhausen, Germany) were filled with 170 μl sample and 10 μl Silica particles (Merck, Darmstadt, Germany) and transferred to a Biomek® 2000 Workstation (Beckman Coulter, Fullerton, CA, USA). One percent Sarkosyl was added, and the plate was incubated at 65 °C for 10 min. and at room temperature for 10 min. The supernatant was removed, and the paramagnetic beads were washed twice with 50% ethanol. DNA was eluted from the silica particles by suspension of the particles in 100 μl Buffer C (1 mM EDTA pH 8.0, 10 mM Tris–HCl pH 8.0) at 65 °C for 30 min. The adequacy of the automated DNA extraction procedure was evaluated by repeating the DNA extraction in 20 samples using the modified MoBio 96-well manual extraction method adopted by the Earth Microbiome Project. (Caporaso et al. 2012) The samples gave very similar results regardless of DNA extraction method used.

*PCR*

1 ul DNA extracted from fecal samples was amplified by PCR reactions by 16S rRNA specific primers (515F-806R) (http://www.earthmicrobiome.org/emp-standard-protocols/16s/). All reactions were set up as 25ul samples in 96 well Thermo-fast 96, low profile, 0,2ml, non-skirted PCR plates (ABgene Thermo scientific, UK) with Cas1200 Corbett robot (Qiagen). 10ul HotMastermix enzyme (5PRIME GmbH, Germany), 0,2uM forward-/ reverse primers (ILHS_515fa/ IL_806rcbc) and 13ul PCR grade water (Qiagen) were used.

*Sequencing and data processing*

Sequencing of the V4 region of the 16S rRNA gene using the Illumina HiSeq instrument. To control for variation in sequencing effort, the data were rarified at a depth of 20000. We used a recently developed sub-operational-taxonomic-unit (sOTU) approach, Deblur, which uses error profiles to obtain putative error-free sequences from Illumina sequencing platforms(Amir et al. 2017). By removing noise, Deblur gives a higher resolution than OTU-based analyses or analyses of raw sequence data, and because it is reference free, it may pick up sequences of novel bacteria that are not represented in existing databases. Data processing was performed in the Quantitative Insights Into Microbial Ecology (QIIME) pipeline version 1.9.1. (Kuczynski et al. 2005).

# References

Amir A, McDonald D, Navas-Molina JA, Kopylova E, Morton JT, Zech Xu Z, et al. 2017. Deblur rapidly resolves single-nucleotide community sequence patterns. mSystems 2.

Caporaso JG, Lauber CL, Walters WA, Berg-Lyons D, Huntley J, Fierer N, et al. 2012. Ultra-high-throughput microbial community analysis on the illumina hiseq and miseq platforms. The ISME journal 6:1621-1624.

Kuczynski J, Stombaugh J, Walters WA, González A, Caporaso JG, Knight R. 2005. Using qiime to analyze 16s rrna gene sequences from microbial communities. In: Current protocols in microbiology:John Wiley & Sons, Inc.
